# Rational design of chimeric Multiepitope Based Vaccine (MEBV) against human T-cell lymphotropic virus type 1: An integrated vaccine informatics and molecular docking based approach

**DOI:** 10.1371/journal.pone.0258443

**Published:** 2021-10-27

**Authors:** Muhammad Hamza Tariq, Rashid Bhatti, Nida Fatima Ali, Usman Ali Ashfaq, Farah Shahid, Ahmad Almatroudi, Mohsin Khurshid

**Affiliations:** 1 Atta ur Rehman School of Applied Bioscience, National University of Science and Technology, Islamabad, Pakistan; 2 Center of Excellence in Molecular Biology, University of the Punjab, Lahore, Pakistan; 3 Department of Bioinformatics and Biotechnology, Government College University Faisalabad, Faisalabad, Pakistan; 4 Department of Medical Laboratories, College of Applied Medical Sciences, Qassim University, Buraydah, Saudi Arabia; 5 Department of Microbiology, Government College University Faisalabad, Faisalabad, Pakistan; University of Balochistan, PAKISTAN

## Abstract

Human T-cell lymphotropic virus type 1 (HTLV-1) is an infectious virus that has been linked to adult T cell leukemia /lymphoma, aggressive CD4-T cell malignancy and many other immune-related medical illnesses. So far, no effective vaccine is known to combat HTLV-1, hence, the current research work was performed to design a potential multi-epitope-based subunit vaccine (MEBV) by adopting the latest methodology of reverse vaccinology. Briefly, three highly antigenic proteins (Glycoprotein, Accessory protein, and Tax protein) with no or minimal (<37%) similarity with human proteome were sorted out and potential B- and T-cell epitopes were forecasted from them. Highly antigenic, immunogenic, non-toxic, non-allergenic and overlapping epitopes were short-listed for vaccine development. The chosen T-cell epitopes displayed a strong binding affinity with their corresponding Human Leukocyte Antigen alleles and demonstrated 95.8% coverage of the world’s population. Finally, nine Cytotoxic T Lymphocytes, six Helper T Lymphocytes and five Linear B Lymphocytes epitopes, joint through linkers and adjuvant, were exploited to design the final MEBV construct, comprising of 382 amino acids. The developed MEBV structure showed highly antigenic properties while being non-toxic, soluble, non-allergenic, and stable in nature. Moreover, disulphide engineering further enhanced the stability of the final vaccine protein. Additionally, Molecular docking analysis and Molecular Dynamics (MD) simulations confirmed the strong association between MEBV construct and human pathogenic immune receptor TLR-3. Repeated-exposure simulations and Immune simulations ensured the rapid antigen clearance and higher levels of cell-mediated immunity, respectively. Furthermore, MEBV codon optimization and *in-silico* cloning was carried out to confirm its augmented expression. Results of our experiments suggested that the proposed MEBV could be a potential immunogenic against HTLV-1; nevertheless, additional wet lab experiments are needed to elucidate our conclusion.

## Introduction

The Human T-cell lymphotropic virus 1 (HTLV-1) is a part of family “Retroviridae”, subfamily “*Orthoretrovirinae*” and genus “Deltaretrovirus” [1]. It is the first human retrovirus to be ever reported, having been identified independently in the United States [2] and Japan [3] in 1980 and 1982 respectively. HTLV-1 contains an outer envelope that encloses single stranded, segmented, and positive-sense RNA genome. It is an infectious virus that has infected nearly 15 to 20 million people globally [4, 5], however, the true number of affected individuals is undetermined, due to inadequate epidemiological studies in the endemic areas [4]. HTLV-1 is widespread in the United States, South America, Africa, Oceania, Caribbean and Japan [2, 6–8]. Increased age, polygamy and insecure sex are the potential risk factors of HTLV-1 infection, whereas, transmission within humans occur due to injecting drugs via parenteral routes, blood transfusion and breastfeeding [9–11].

HTLV-1 encompasses 8507 nucleotides in its genome, which like all other retroviruses encodes both structural (such as gag and core antigens) and enzymatic proteins (such as Reverse Transcriptase). Furthermore, being a complex retrovirus, HTLV-1 also employs internal initiator codons and an alternative splicing procedure, to synthesize several accessory and regulatory proteins. It has four Open Reading Frames (ORFs), where ORF-3 and ORF-4 are the most important ones as they codes for Rex protein and Tax transactivating protein respectively [12, 13]. Rex is required for regular transportation of viral RNA [14, 15] and Tax triggers various transcription factors and enhancers to initiate transcription of many cellular genes that are primarily linked to host cell proliferation [16, 17].

Almost 90% of the HTLV-1 affected individuals show no symptoms throughout their lives, however, almost 10% patients establish a chronic inflammatory disease, among which Adult T-cell leukemia (ATL) is the most prevalent one, found in 5% of the HTLV-1-infected people [18, 19]. HTLV-1-associated myelopathy/tropical spastic paraparesis (HAM/TSP) is the other secondary complication that is prevalent in about 1–4% patients of HTLV-1 [20]. Other HTLV-1 complexities include; HTLV-associated uveitis [21], Leprosy [22], aggressive CD4-T cell malignancy [23], Tuberculosis [24], Strongyloides stercoralis [25].

The mechanism of how HTLV-1 mediates the development of these disorders is not evidently known, however, it is believed that these disorders are a probable result of the virus’s potential to elicit lymphocyte activation. HTLV-1 infects cytotoxic T cells (CD8^+^) as well as Helper T cells (CD4^+^), consequently, the immune system triggers a T-cell mediated immunity, which binds to MHC-I or MHC-II complexes. Activated T-cells prompts the secretion of cytokines, whose major role is to trigger a rapid immune response against HTLV-1 [26–30]. In actuality, the immunopathogenesis of HTLV-1 is worth noting, as its lifetime persistence in T-cell lymphocytes result in enhanced interaction amongst the immune system and virus, which ultimately results in a number of HTLV-1 associated diseases. Such complications are either due to the direct viral action on immune system or an outcome of the immune reactions against virus. Till now, no effective vaccine against HTLV-1 is known; however, number of affected individuals is continuously increasing.

The rise of immunoinformatics and bioinformatics has resulted in great advancements in vaccine design and development. Methods like Structural vaccinology and reverse vaccinology have tremendously increased the development of new vaccines against pathogens [31]. Owing to these easily accessible bioinformatics tools, we can precisely predict the antigenicity of coveted proteins. Accurate determination of such antigenic components is essential to develop an effective vaccine subunit [32].

Investigation of possible epitopes and designing of Multi-Epitope Based Vaccine (MEBV) is an important development in vaccinology as they can proficiently evoke both humoral- and cell-mediated immune responses [33, 34].

In contrast to conventional vaccines, MEBVs hold a number of merits, including: their affordability, harmlessness and their efficiency in engineering the epitopes rationally [35]. Consequently, the present study was intended to use immunoinformatics techniques to design a MEBV against HTLV-1. The whole proteome of HTLV-1 was considered to forecast an effectual MEBV. Molecular docking analysis was also carried out to examine the binding potential and stability of designed MEBV with human pathogenic receptors. Besides authenticating the immunogenic potential of the designed MEBV, *in- silico* immune simulations were also executed. Finally, the MEBV codon optimization was accomplished for *E*. *coli system*, followed by *in-silico* cloning to validate the profiling expression.

## Methodology

**Fig 1:**
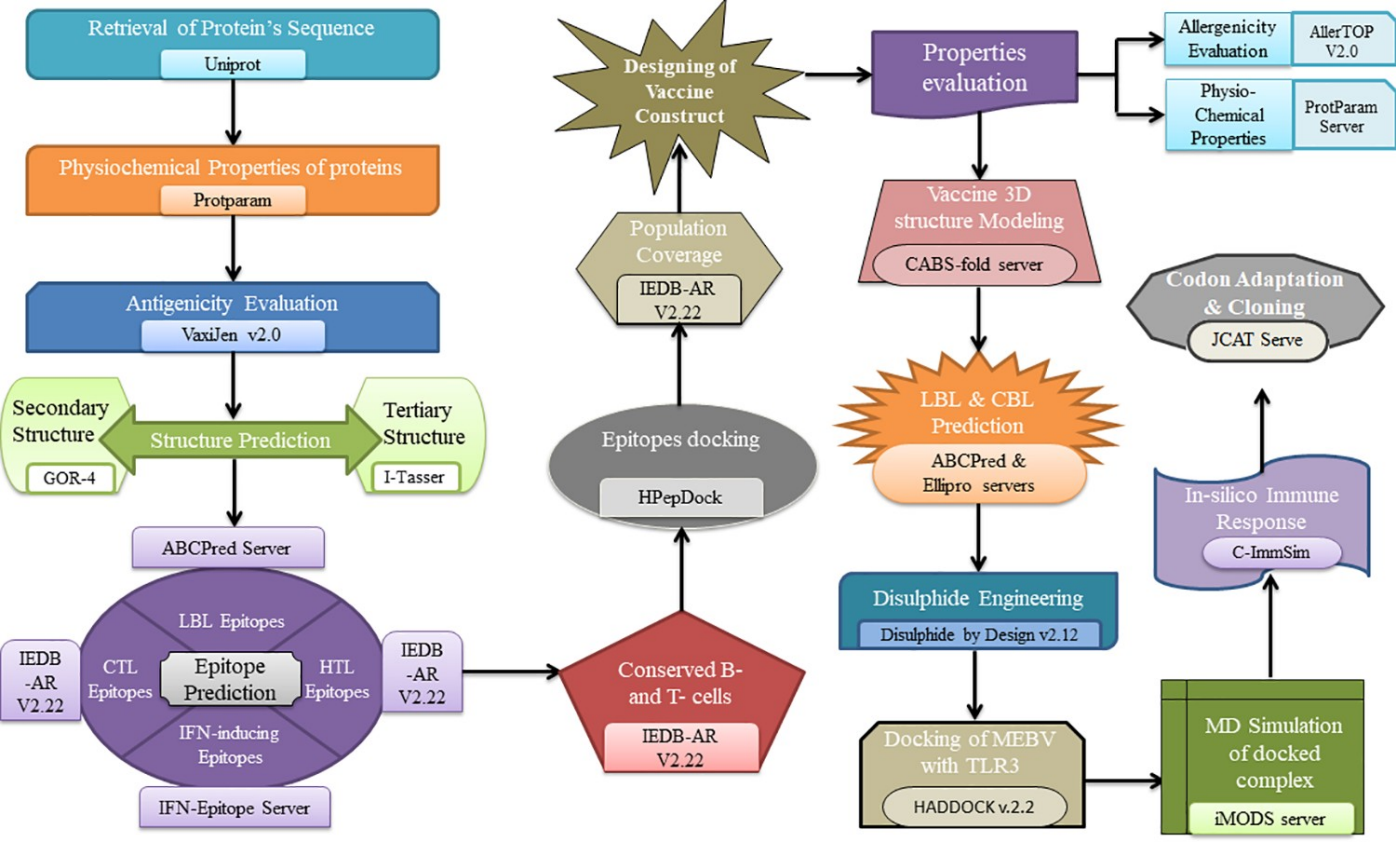
Diagrammatic depiction of strategy adopted in the current study.

Flow diagram of the entire procedure opted in the current research work is illustrated in Fig 1.

### Retrieval and analysis of HTLV-1 proteome

Firstly, the proteome of HTLV-1 was assessed and downloaded from the Uniprot database. Vaxijen v.2.0 online website was operated to find the proteins having strong antigenicity. This tool evaluates antigenic properties by virtue of alignment-free method and chiefly by analyzing their physio-chemical properties [36]. Fasta sequences were pasted as an input sequence whilst 0.4 value was set as a threshold. Proteins depicting strong antigenicity were then estimated for their physiochemical properties through the ProtParam tool of ExPasy online server. This software only analyzes the physiochemical properties depending upon the submitted protein sequences, however, this online tool is unable to identify the post-translational modifications of protein [37]. Finally, proteins showing acceptable antigenicity and physio-chemical properties were tested for similarity index with human proteins; Blastp was utilized for this purpose. Proteins showing similarity index more than 37% were excluded for further experimentation. The 3D structures of shortlisted proteins were recovered from the Protein Data Bank (PDB) database [38], however, 3D structure of the proteins for which no structure was found in PDB was predicted by online bioinformatics tools. Secondary structure was investigated via GOR4 server [39], whereas tertiary structure was modeled through I-TASSER. I-TASSER is a classified bioinformatics tool that is actually a structure modeling server used for analyzing the tertiary structure by using ab-initio modeling [40]. Predicted 3D structures were then refined through GalaxyRefine2 server [41], followed by quality assessment through Rampage and PROSA web servers where former one assesses the structure by Ramachandran plot and later one evaluates it by measuring the z-score [42].

### T-cell epitope recognition and evaluation

Forecasting of T-cell epitopes (TCEs) not only makes the whole procedure of vaccine designing cost-effective but also, in compared to the laboratory experiments, lessens the time required for the entire protocol [43]. The Immune Epitope database Analysis Resource (IEDB-AR) version 2.22 [44] was employed to recognize the TCEs. This online server provides many tools for the estimation and assessment of antigenic epitopes, MHC-I binding prediction tool (http://tools.immuneepitope.org/mhci/) made it possible to identify 12-mer MHC-class I epitopes (Cytotoxic T-lymphocytes). MHC-II binding prediction tool was utilized to recognize 15-mer MHC-class II T-cell epitopes (Helper T-lymphocytes). Fasta formatted protein sequences were provided, consensus mode was elected as prediction technique, human was chosen as source specie, and all offered alleles were considered for the estimation of epitopes. Lower consensus score accounts for the strong binding capacity to alleles, therefore, epitopes with consensus score lower than 2 were taken for further experiments. All of the predicted epitopes were further analyzed through different online bioinformatics tools i.e. VaxiJen v2.0 was employed to evaluate antigenic character, IEDB-AR v.2.22 MHC-I immunogenicity tool [45] was utilized for identifying immunogenicity, AllergenFP v1.0 server [46] was used for analyzing allergenic character and Toxin Pred online server [47] was engaged to forecast the toxic behavior of predicted epitopes. AllergenFP server computes descriptor-based alignment-free fingerprint protocol to identify the allergenic character of peptides with 88.9% accuracy [46], whereas, Toxin Pred online tool engages machine-learning with a quantitative matrix to predict toxicity [47].

### B-cell epitope recognition and evaluation

Recognition of B-Cells Epitopes (BCEs) is a vital step in the designing of MEBVs as it releases antibodies that lead to the generation of humoral immunity. There are two different types of BCEs; namely, linear BCEs and conformational BCEs. Linear BCEs were identified by the means of ABCPred online tool, that uses the neural networking based approach to identify the BCEs [48]. The amino acid length and the threshold values were set to 14 and 0.5 respectively. Additionally, conformational BCEs were predicted through ellipro tool of IEDB-AR v.2.22. Ellipro working is based on geometric properties of protein structure such as adjacent cluster residues, entire protein shape and residual protrusion index [49], therefore, protein structures were uploaded in PDB format whereas all other parameters were set as by default. Finally, the selected BCEs were evaluated for antigenic, allergic, immunogenic, and toxic profiles through vaxiJen v2.0, AllergenFP v1.0, IEDB-AR v.2.22 MHC-I immunogenicity and ToxinPred respectively.

### Conservation analysis and selection of predicted epitopes

An epitope should demonstrate 100% conservancy if it has to be used for vaccine designing protocol, therefore, conservancy of the estimated BCEs and TCEs was determined through Epitope Conservancy Analysis tool [50], offered by IEDB-AR v.2.22. Epitopes showing 100% conservancy were considered for further study. Forecasting of cytokine-prompting abilities of epitopes is an important parameter in finding out the efficacious epitopes that could be used for vaccine designing. Interferon-gamma (IFN-γ) is a chief cytokine that can prompt intrinsic safe responses and also restrict viral replication [51]. Besides that, IFN-γ also promotes the flexible immune responses by triggering both Helper T Lymphocyte (HTL) and Cytotoxic T Lymphocyte (CTL). The IFN-γ inducing probability of the forecasted epitopes was examined through IFNepitope online tool that works on MERCI and support vector machine hybrid algorithms. Eventually, epitopes were filtered after overlapping, toxicity, antigenicity and conservancy tests, were chosen for subsequent examination.

### Epitope modeling and molecular docking

PEP-FOLD v.3.0 online tool was considered to construct the 3D model of short-listed HTL and CTL epitopes from scratch. sOPEP sorting scheme with 200 simulations was adopted for this purpose. PEP-FOLD v.3.0 estimates the peptide’s tertiary structure by implementing forward backtrack/taboo sampling algorithm [52]. Once the 3D structures of epitopes were ready, they were docked with particular Human Leukocyte Antigen (HLA) alleles to assess their binding affinity. Crystal structures of HLAs were downloaded from RCSB PDB [38]. Docking was executed through HPepDock server [53] and the docked complex was inspected via PyMOL molecular graphic system v.1.3 [54].

### Estimation of population coverage

Expression of the distinctive HLA provide astounding dispersion at varying frequencies, that are assorted in different regions and ethnicities all over the Globe [55]. Thus the allele dispensation plays an essential part in the development of an efficacious MEBV [56]. In this research work, the population coverage analysis tool [57] of IEDB-AR v.2.20 was used to examine the population coverage of the short-listed HTL and CTL epitopes and their particular HLA-binding alleles.

### Designing of vaccine construct

Generally, epitopes that are highly antigenic, 100% conserved, non-allergic, with noteworthy population coverage, having no identity with human proteins and exhibiting significant binding capability with human HLA allele are considered to construct a MEBV. Hence in the current study, only those epitopes were considered for vaccine designing that were displaying the above-mentioned properties. β-defensin adjuvant was connected to the very first CTL epitope via EAAAK linker, the purpose of which is to enhance the immune response. β-defensin was employed as an adjuvant owing to its capability of acting as an antimicrobial as well as an immunomodulatory agent [58]. All other CTL, HTL and LBL epitopes were joined with each other through AAY, GPGPG and KK linkers correspondingly. The purpose of connecting them with these linkers is to support their individualistic immunogenic actions.

### Host homology analysis

Homology to host protein can prompt autoimmunity, therefore, the NCBI BLASTp online software was opted to examine the homology of constructed MEBV with the entire human proteome, [59]. Vaccine’s primary sequence was added in the FASTA format. The target organism under consideration was *Homo sapiens* (taxid:9606).

### Evaluation of allergenicity, antigenicity and physiochemical properties

Different online tools were adopted to evaluate the final vaccine construct for physiochemical properties, antigenicity and allergenicity. VaxiJen v2.0 online web tool was accessed to scrutinize the antigenic behavior of MEBV construct, whereas AllerTop v2.0 was adopted to evaluate its allergenicity. AllerTop includes the alignment-free estimation of the allergenic character of a protein by assessing its chief physicochemical properties [60]. Protparam tool of Expasy was used to recognize different physiochemical effects of the final MEBV protein for instance; theoretical pI, Grand Average of Hydropathicity (GRAVY), aliphatic index, stability index, molecular weight and expected half-life for mammal cells, yeast and *E*. *coli*. Additionally, solubility on the subject of overexpression in *E*. *coli* cells was anticipated through SOLpro tool [61] of SCRATCH suite.

### Secondary structure of MEBV

Secondary structure is a major contributing factor of protein-folding characteristics; MEBV structure was designed using the Psipred server. This server considers the two feed-forward neural networks, where one network refines the other through the medium of Position-Specific Iterated–BLAST [62].

### Tertiary structure of MEBV

The final constructed MEBV is the combination of distinct epitopes therefore no appropriate template was available, hence its tertiary structure was identified by following de novo modeling approach by employing CABS-fold server. This server works on CABS modeling approach in addition to multiscale modeling pipeline and Replica Exchange Monte Carlo pattern [63]. Predicted 3D structure was then refined by GalaxyRefine2 server. The quality of the modified structure was then checked through RAMPAGE server, ProSA-web server, ERRAT server and Verify 3D server. RAMPAGE server evaluates the 3D structure based on Ramachandran Plot analysis [64], ProSA-web server evaluates structure’s validity based on the statistical assessment of protein structures [42], ERRAT server identifies the structure’s excellence by calculating the non-bonded connections in the examined structure [65] and Verify 3D estimates the agreement between the tertiary structure and its own amino acids sequence [66].

### Prediction of B-cells epitopes

Both linear BCEs and conformational BCEs of constructed MEBV were distinguished using the ABCPred online server and Ellipro tool of IEDB-AR v.2.22. In the former, MEBV primary sequence was provided as the input and the amino acid length was fixed at 14, while in the latter one all of the parameters remained unchanged and the MEBV tertiary structure was inserted as input. PyMOL molecular graphic system v.1.3 was again adopted to demonstrate the discontinuous epitopes in the final designed MEBV [54].

### Protein-protein docking between vaccine and immune receptor (TLR3)

Interactions between the vaccine protein and the host immune cells trigger an efficient immune reaction. Therefore, molecular docking approached was used to discern the binding capability of MEBV with human immune receptors. TLR3 (PDB ID: 1ZIW) was selected for this study as the protein is known for eliciting an efficient antiviral immune response. Docking of the MEBV with TLR3 was performed using the HADDOCK v.2.2 server [67], that involves an information-driven flexible docking approach, useful for studying biomolecular complexes [68]. Visualization of the docked complex was carried out through the PyMOL molecular graphic system v.1.3 [54] and the interacting residues were identified by analyzing the structures in PDBsum online database [69].

### Disulphide engineering of final vaccine construct

Disulphide engineering is a new method of adding disulphide bonds between amino acids of a protein structure. A disulphide bond is the covalent bond that imparts significant stability to a protein structure by authorizing the accurate geometric conformations. Disulphide by Design v2.12 online tool was employed for disulphide engineering of MEBV protein. Refined MEBV structure was submitted and run for the residue-pair examination, potential residue pairs were identified for mutation, and cysteine residue was taken as an ultimate objective [70]. Residues having an energy score lesser than 2.2 kcal/mol and χ^3^ angle within a range of − 87° and + 97° were considered for the formation of disulphide linkages [70].

### *In-silico* estimation of vaccine construct

*In-silico* immune simulation was achieved through C-ImmSim 10.1 server to substantiate the immunological responses of the designed MEBV. The C-ImmSim activates three chief constituents of the functional mammal physiological system, including the bone marrow, lymph node, and thymus, [71]. The parameters entered for running the software were the following: volume (10), HLA (A0101, A0101, B0702, B0702, DRB1_0101, DRB1_0101), number of steps (100), random seed (12345), number of injections set to 1. All other parameters were set to be unchanged.

### *In-silico* codon optimization and cloning

Once the properties and *in silico* immune simulations of constructed MEBV were carefully analyzed, codon optimization was carried out by using Java Codon Adaptation Tool [72]. Codon optimization is a procedure commonly utilized to ameliorate the gene expression and enhance the translational efficiency of a gene of interest by inserting codon bias of the host organism [73]. Codon optimization of MEBV was done in E. coli K12 prokaryotic expression system. Three extra available options were chosen to dodge: (1) restriction enzymes cleavage sites (2) prokaryote ribosome binding-site and (3) rho-independent transcription termination. GC content and Codon adaptation index (CAI) values were noted and checked whether they fall under the acceptable range or not, which was 30–70% for GC content and <0.8 for CAI [74]. Besides that, restriction and cloning of the modified MEBV sequence was expedited by the addition of BamHI and HindIII restriction sites at its C and N-terminals correspondingly. This modified sequence was then taken for *in-silico* cloning via SnapGene v4.2 tool. Cloning was done in vector E. coli pET30a (+) to validate the expression of sequence in in vitro systems.

### Molecular Dynamics (MD) simulation

MD simulation is contemplated as a principle methodology to scrutinize the stability of docked complexes [75]. These simulations provide important supporting information for predictions and interpretations of the experimental data. MD simulations were performed through GROMACS 5.0 Software [76, 77]. In carrying out the simulations, for the docked system of MEBV -TLR3 and the structure of TLR3, default parameters were used. The systems were solvated in a cubic water box, with the complexes placed at least 1.0nm from the edge of the box. The Optimized Potential for Liquid Simulation (OPLS) force field [78] was employed and the physiological pH of the system was maintained using the Single Point Charge Extended (SPCE) water model [79]. The neutrality of the systems was maintained by adding Na+ or Cl- ions with the steepest descent approach for energy minimization comprising of 50,000 cycles. The systems were then equilibrated for the constant number of particles, volume and temperate (NVT) and constant number of particles, pressure and temperature (NPT), following these equilibrations, the systems were simulated for 20ns. The trajectories for the complex and the protein were saved after every 2 fs.

## Results

### Proteome retrieval and highest antigenic protein selection

Full proteome of HTLV-1 was downloaded from Uniprot (Proteome ID: UP000007683) HTLV-1 proteome consisted of nine different proteins. Proteins names and accession no are enlisted in S1 Table. Antigenicity of all of these proteins was evaluated by VaxiJen v.2.0, results of which represented that only six proteins had vaxijan score >0.45 therefore, the sequence of only these proteins was submitted to Blastp for identifying their similarity with human proteins. Blastp results showed that Accessory Protein p12I (AP) and Protein TAX-1 (PT) had no significant similarity with any of the human proteins whereas Envelop Glycoprotein gp 62 (GP) depicted a maximum similarity index of 32.14% with Syncytin-1 Precursor (Accession No: NP_001124397.1) while the other 3 proteins had a similarity index of more than 60% with one or more human proteins (S1 Table), hence AP, PT and GP were selected for further study. Moreover, these three proteins were subjected to Protparam online tool to compute their physical properties including aliphatic index, theoretical pI, stability profiling, molecular weight and half-life (S2 Table). Secondary structure of the proteins was appraised via GOR4 server (S3 Table). Crystal structure of short-listed proteins was searched in RCSB PDB database, from where only the 3D structure of GP was found (PDB ID: 1mg1.1.A) whilst no structure was found for other two proteins, therefore, their structures were predicted by i-TASSER online tool and refined by the GalaxyRefining2 server (S1 Fig). The attributes of these final structures were elucidated through Ramachandran plot assessment, ERRAT Value and z-score. (S2 Fig and S4 Table) depicts that both of the predicted 3D structures had a good quality as all of the analyzed parameters were within the acceptable range. The purpose of this designed model was to estimate the conformational BCE of chosen proteins.

### T-cell epitope recognition and evaluation

IEDB consensus method was adopted to forecast TCEs (including both MHC-I and MHC-II) of target proteins. Owing to their strong defensive capabilities, epitopes showing interactions with more than one allele are taken as the most appropriate epitopes. Conservancy of predicted epitopes inside the protein sequences was determined through IEDB conservancy analysis tool. Their allergenicity and antigenicity was evaluated by Allergen FP 1.0 and Vaxijen. Epitopes that are highly antigenic, bound to multiple alleles, 100% conserved, and non-allergenic were taken for further study. In total, 40 CTL epitopes (AP-07, PT-11 and GP-22) (S5 Table) and 39 HTL epitopes (AP-18, PT-10 and GP-11) were shortlisted (S6 Table).

### B-cell epitope recognition and evaluation

Linear/continuous B cell epitopes (LBL) of all target proteins were picked by ABCPred server. Just like T-cell epitopes, selected LBL were 100% conserved, non-allergenic and antigenic. In accordance with this criterion, 17 LBL epitopes (AP-1, GP-12, and PT-4) were predicted (S7 Table). Whereas, Ellipro online tool was adopted to identify conformational/discontinuous B cell epitopes (CBL) by considering the 3D models of target proteins and a total of 8 CBL epitopes (AP-3, GP-3, and PT-2) were predicted in this way (S8 Table).

### Evaluation and selection of epitopes for further analyses

The criteria intended to include the epitopes in the MEBV was that they should be 100% conserved among proteins, should be significantly antigenic/immunogenic, should be non-toxic and non-allergenic, should generate IFN-γ response and should not be within the glycosylation sites and post-translation modification sites of the particular protein. Therefore, epitopes satisfying the above-mentioned criteria were selected for further analyses. In total, 9 CTL epitopes (AP-3, GP-4 and PT-2), 6 HTL epitopes (AP-2, GP-2, and PT-2) (Table 1) and 5 LBL epitopes (AP-1, GP-3 and PT-1) were selected to construct MEBV (Table 2).

**Table 1:** Final selected T-cell epitopes from HTLV-1 antigenic proteins employed to develop the MEBV construct and their binding details with their corresponding HLA alleles.

Sr. No	Epitopes	Protein	Position	Antigenicity	Immunogenicity	Alleles	Binding Score (kcal/mol)
MHC Class I							
1.	LSPLALTALLLF	AP	9–20	1.0379	0.06216	HLA-B*57:01	-144.82
2.	LPITMRFPARWR	AP	72–83	1.1198	0.27479	HLA-B*57:01	-187.26
3.	FPARWRFLPWKA	AP	78–89	1.4942	0.48458	HLA-B*27:05	-185.32
4.	PYWKFQHDVNFT	GP	131–142	1.1361	0.011	HLA-C*07:02	-178.38
5.	HLTLPFNWTHCF	GP	266–277	1.0691	0.34638	HLA-B*57:01	-187.47
6.	YAAQNRRGLDLL	GP	374–385	1.0626	0.02544	HLA-C*06:02	-179.81
7.	LPSRVRYPHYSL	GP	470–481	1.0175	0.00525	HLA-C*07:02	-179.92
8.	QLSPPITWPLLP	PT	158–169	0.7867	0.28116	HLA-B*35:03	-152.39
9.	EYTNIPISLLFN	PT	311–322	1.0096	0.14199	HLA-A*24:02	-143.93
MHC Class II							
10.	PPPAPCLLLFLPFQI	AP	34–48	0.7086	0.06395	HLA-DRB1*01:02	-181.08
11.	PPAPCLLLFLPFQIL	AP	35–49	0.6695	0.09924	HLA-DRB1*01:02	-173.42
12.	TNYTCIVCIDRASLS	GP	221–235	1.0023	0.19499	HLA-DRB1*03:06	-180.28
13.	NYTCIVCIDRASLST	GP	222–236	0.9098	0.0536	HLA-DRB1*03:06	-171.90
14.	GDCVQGDWCPISGGL	PT	21–35	1.1352	0.14051	HLA-DRB1*03:05	-152.31
15.	CVQGDWCPISGGLCS	PT	23–37	1.1086	0.16304	HLA-DRB1*03:05	-150.82

(* is the part of allele’s specific naming system).

**Table 2:** Final chosen LBL epitopes from HTLV-1 antigenic proteins employed to develop the MEBV construct.

Sr. No	LBL Epitopes	Protein	Position	Antigenicity	Immunogenicity
**1**	PCLLLFLPFQILSG	AP	38	0.6293	0.0873
**2**	TNYTCIVCIDRASL	GP	221	1.0062	0.23894
**3**	LTLPFNWTHCFDPQ	GP	267	1.2029	0.44788
**4**	RRGLDLLFWEQGGL	GP	379	0.9600	0.3664
**5**	HQITWDPIDGRVIG	PT	52	0.8070	0.65428

### Molecular docking between epitopes and HLA alleles

**Fig 2:**
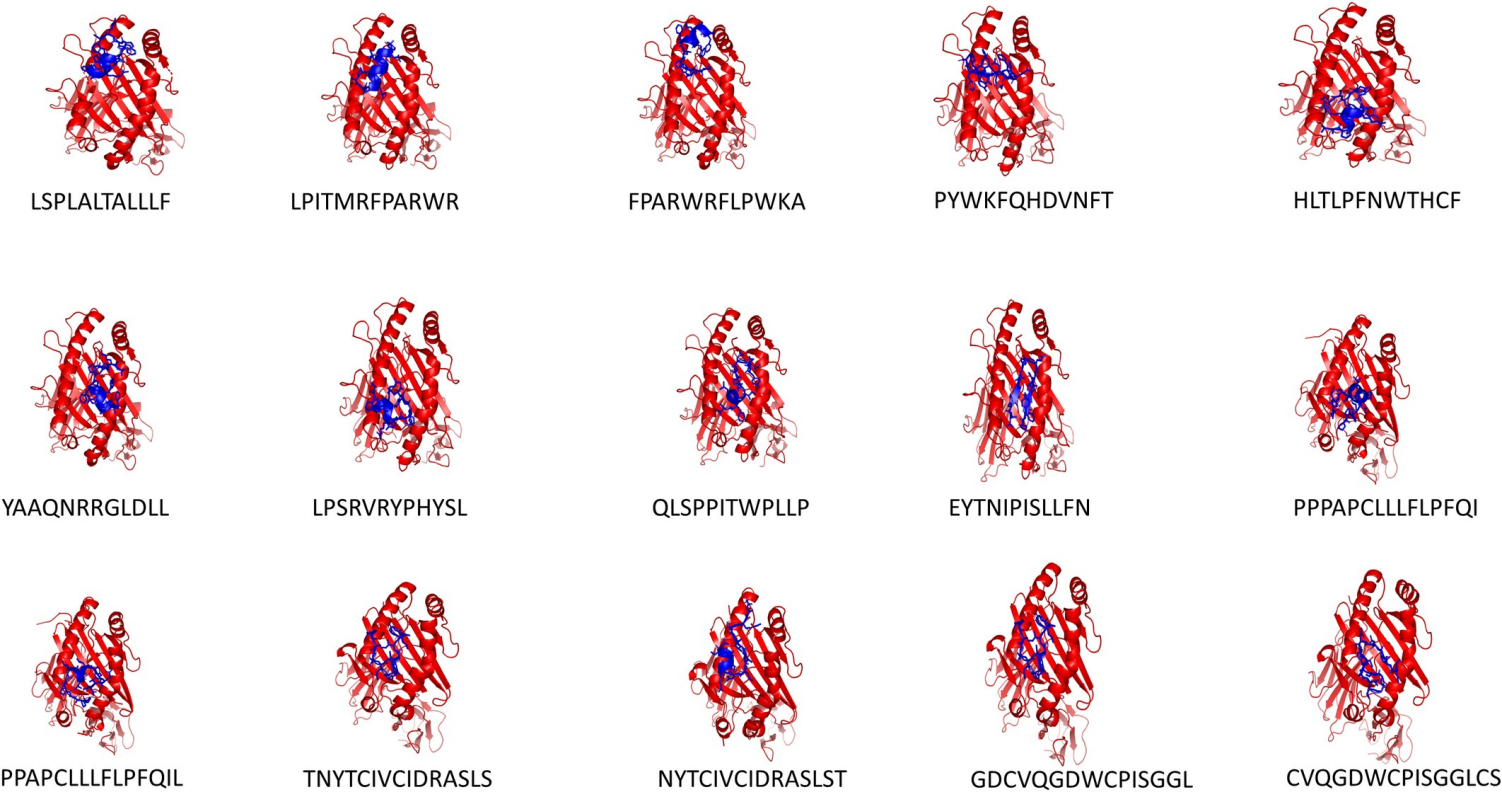
Docked complex of chosen 15 epitopes (blue licorice illustration) and their corresponding HLAs (red cartoon illustration) as demonstrated in Table 1.

The 3D structures of selected CTL and HTL epitopes were modeled through PEPFOLD online tool (S2 Fig). Molecular Docking was executed by HPepDock server to assess the binding affinity of TCEs with their corresponding HLA alleles. All examined epitopes displayed excellent binding affinity with the receptor binding domain of HLA alleles (**Fig 2**).

### Population coverage estimation

**Fig 3:**
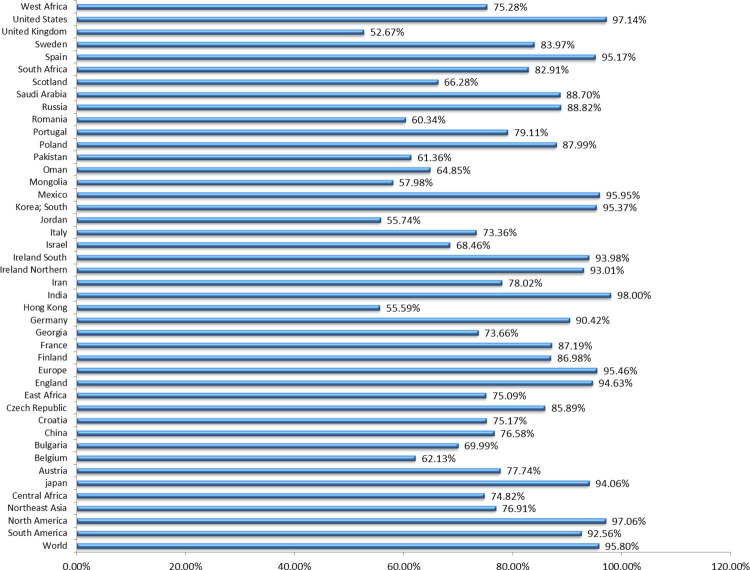
Collective population coverage of the chosen T-cell epitopes based on their corresponding HLA alleles. Areas of specific significance were considered in this display.

The HLA allele distribution varies among different ethnic groups, existing in different geographical regions of the World. This makes the coverage of the population an important parameter in constructing MEBV. This study involves calculation of collective population coverage of shortlisted HTL and CTL epitopes along with their corresponding HLA alleles. This investigation indicated that the selected epitopes have a combined coverage of ~95.80% in the worldwide population (**Fig 3**). Highest population coverage was reported in India (98%), followed by Unites States and Mexico, where 97.14% and 95.95% coverage was reported correspondingly. The results affirmed that selective epitopes are significant for developing the MEBV construct.

### Construction of multi-epitope subunit based vaccine

**Fig 4:**
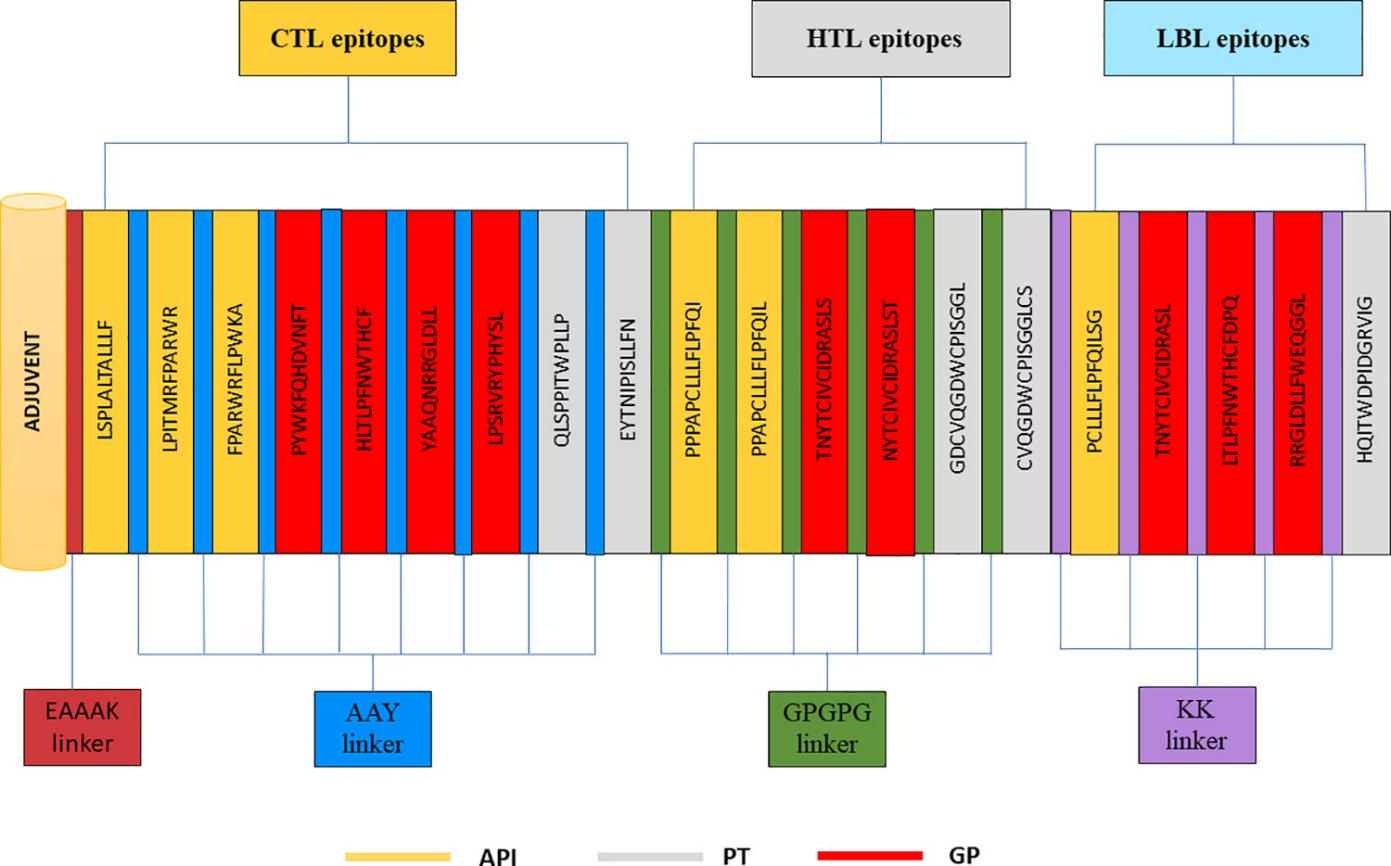
Graphical representation of constructed MEBV structure, encompassing 382 amino acids having one adjuvant (at N-terminal), 9 CTL, 6 HTL and 5 LBL epitopes.

**Fig 5:**
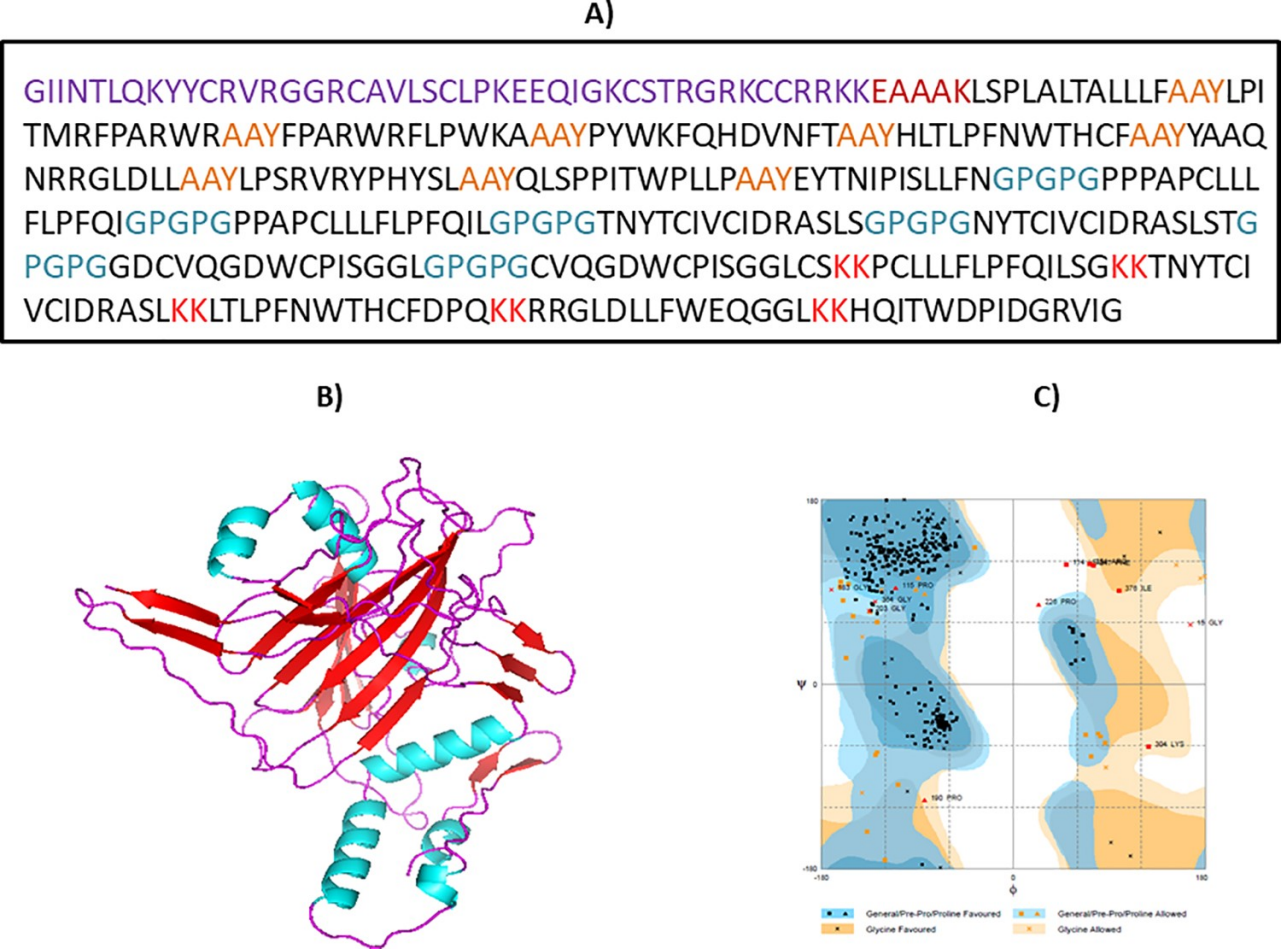
(A) MEBV construct sequence. Black letters represents the epitopes sequences. Purple letters depict adjuvant sequence, Maroon color shows EAAAK linker, Blue color displays GPGPG linkers and red color demonstrates the KK linkers; (B) Pipes representation of refined 3D model of final MEBV construct (cyan color shows ɑ-helix, red color depicts β-strands and magenta color illustrates random loops); (C) Ramachandran plot analysis of estimated MEBV model, showing the presence of 89.2% residues in the favored region.

The MEBV construct was developed by making use of the short-listed epitopes. The HTL, LBL and CTL epitopes were merged with each other using the KK, GPGPG and AAY, KK Linkers correspondingly. The use of linkers not only promotes the process of immunization and epitope presentation but it also inhibits the production of junctional epitopes [80, 81]. Additionally, β-defensin was attached as an adjuvant, to the N-terminal region of the final constructed vaccine through EAAAK linker. This linker adds to the overall stability of the structure while reducing connections with other protein parts by proficient detachment [82]. The final structure of the vaccine construct, displayed in (**Figs 4 and 5A**) comprises of 382 amino acids.

### Immunogenic and physiochemical profiling

The development of a vaccine construct was followed by immunogenic and physiochemical analysis. This was done by evaluating the homology model of MEBV against the human proteome. The results indicated that no significant similarity index was observed between MEBV with any part of the human proteome. Determination of the antigenic, toxic and allergenic properties of the MEBV revealed it to be extremely antigenic, the antigenic score of the complex being 0.7840 (at threshold = 0.5), it was further proved to be potentially non-allergenic and non-toxic in nature. The physiochemical features of MEBV, evaluated using ProtParam online tool showed it had a Molecular weight of 42154.54 Da, while the theoretical pI and GRAVY had values of 9.39 and +0.024, where the positive value indicated hydrophobic nature of the construct. The mean half-life of MEBV was estimated at 30 h > 20 h and >10 h in the mammalian reticulocyte, *E*. *coli* and yeast cells. The solubility of MEBV was calculated using the SOLpro server revealed it was highly soluble, having a probability value of 0.972835. On the basis of these analyses, MEBV was deemed to have strong potential to serve as a vaccine.

### Secondary structure examination

PSIPRED server was accessed to evaluate the secondary structure features of MEBV protein. 18.3% and 14.4% of the total constituent residues contributed to the construction of ɑ-helix and β-strands respectively, whereas the remaining 67.3% amino acids participated in the formation of coils (S3 Fig).

### Tertiary structure modeling

Tertiary structure of MEBV protein was modeled through CABS-fold online tool. Predicted structure was additionally refined through GalaxyRefine2 server (**Fig 5B**). Validity of the final developed model was evaluated using the Ramachandran plot analysis. It indicated 89.2% and 7.6% of the total amino acids residues in the favorable and allowed regions, while only 3.2% amino acids were indicated in the outer region (**Fig 5C**). Based on further evaluation, it was revealed that no poor rotamers existed in the final structure. The probability estimation (Z-Score) and quality factor for the structure had values of -5.53 (S4 Fig) and 55.5944. This refined structure was also passed by verify 3D. All of these findings demonstrated that the refined structure of designed MEBV is of excellent quality.

### Disulphide engineering for vaccine stability

**Fig 6:**
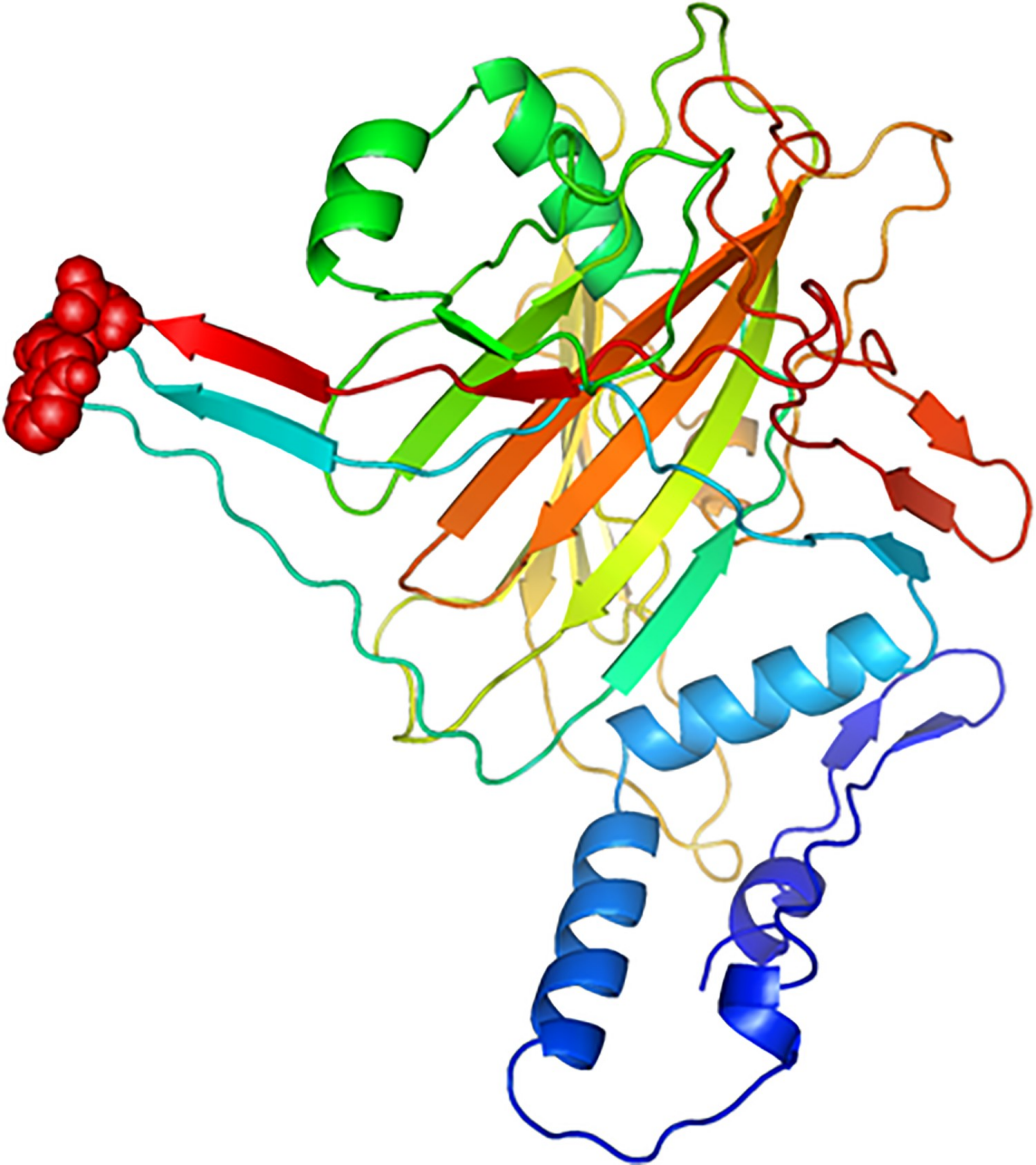
Disulfide engineering, depicting one mutated residues pair with red color.

Disulfide engineering was done via Disulfide by design v2.0 online tool, to improve the stability and constancy of the final refined structure of MEBV. In total, 25 different pairs of residues can be considered for performing disulfide engineering (S9 Table), however, the criteria for the selection of residues pairs is that they must depict the normal Chi3 value and energy. Our designed MEBV demonstrated only single residue pair following the selection criteria, therefore, only two mutations were generated in the selected residue pair i.e. Trp 90-Gly 382, with an energy of 1.42 kcal/mol and Chi3 value of -71.13 (**Fig 6**).

### Estimation of B-cells epitopes in MEBV

**Fig 7:**
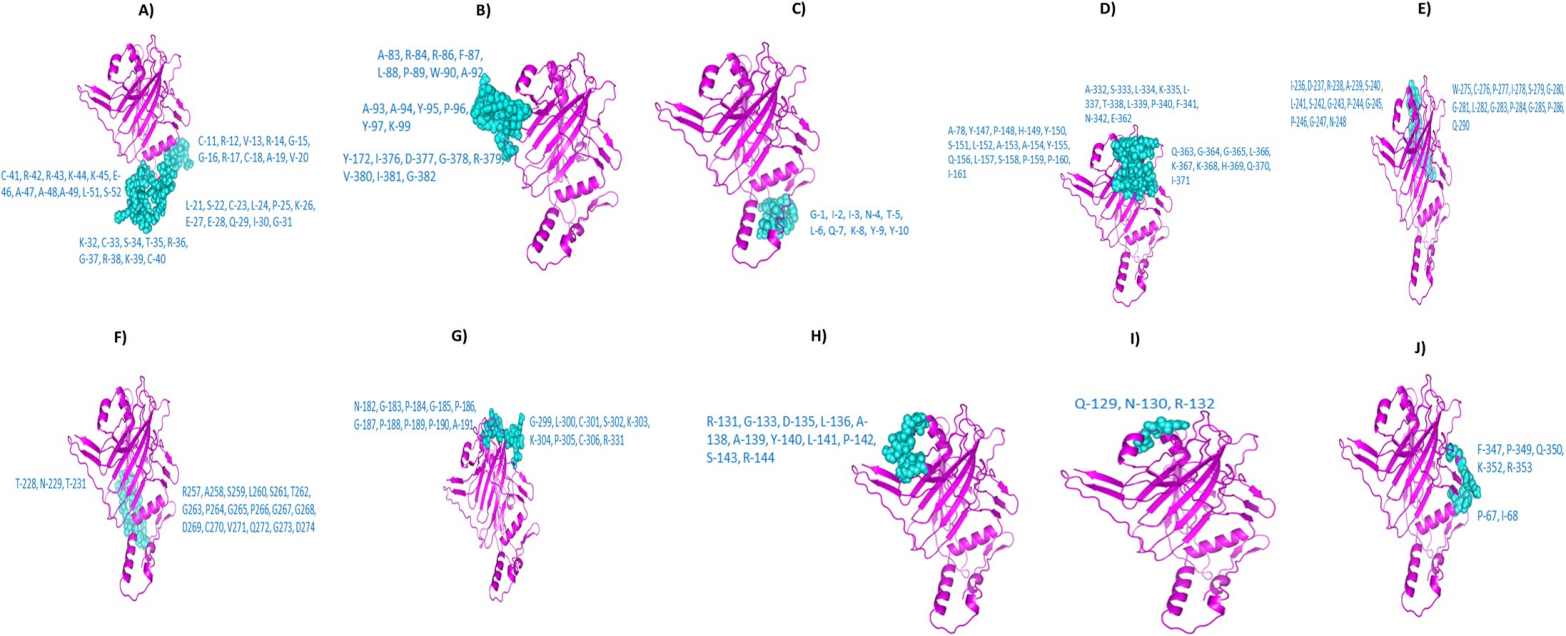
Conformational B-cell epitopes (cyan) recognized in the final MEBV vaccine (magenta).

There are two vital roles which are associated with B-lymphocytes, i.e., antibodies generation and releasing cytokines, where former function is directly involved in inducing humoral immunity. Thus, an ideal MEBV candidate must encompasses B cell epitopes within its final deigned structure. In the current study, ABCPred 2.0 and Ellipro online tools represented that our constructed MEBV encloses thirty-two LBL (S10 Table) and ten CBL epitopes (S11 Table). PyMOL molecular graphic system v.1.3 enabled us to envisage the CBL epitopes in the final structure of MEBV (**Fig 7**).

### Interaction analysis between vaccine and TLR-3

**Fig 8:**
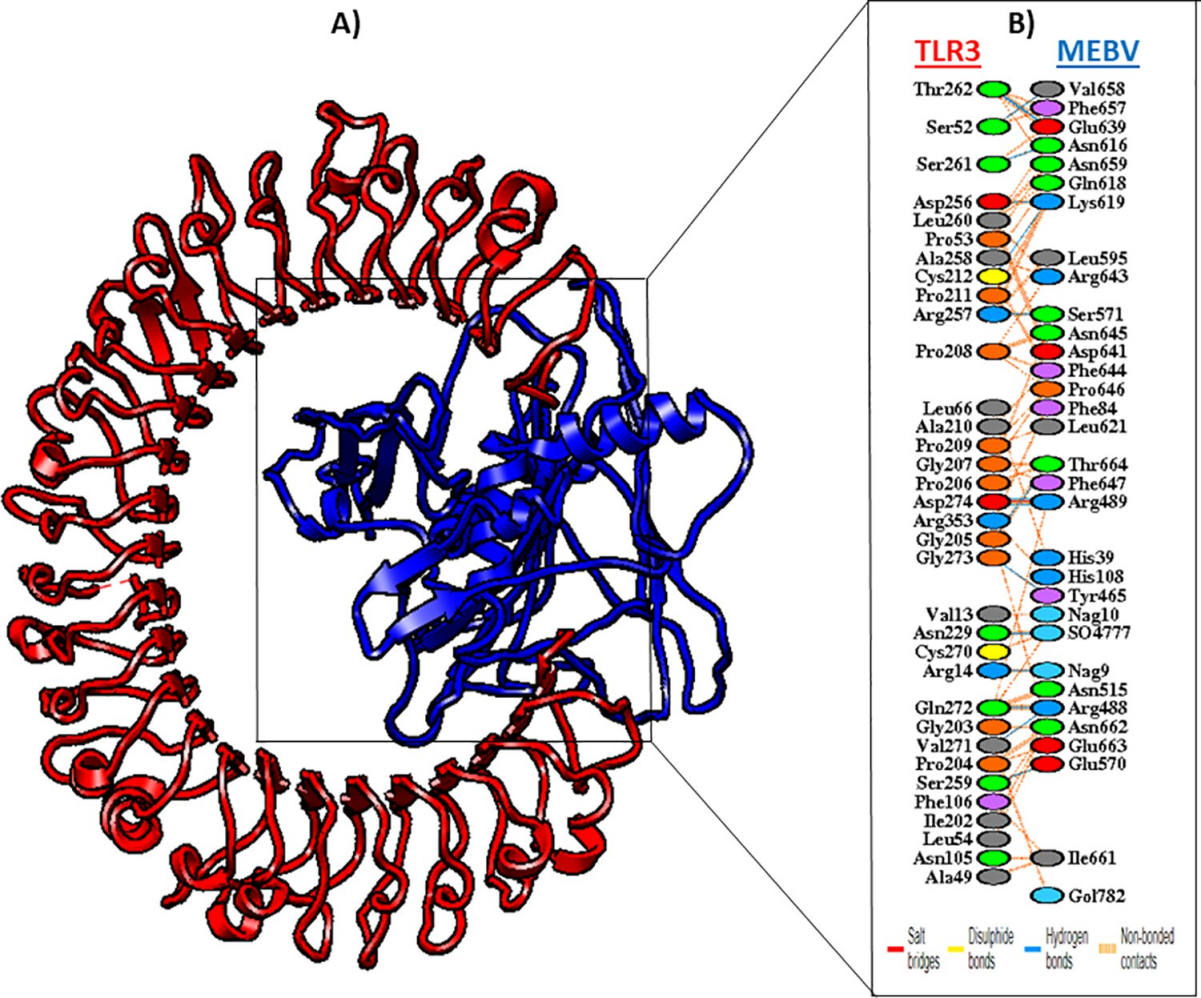
TLR3-MEBV docked complex shown at the left in cartoon representation. Interacting residues of MEBV are highlighted at right side. MEBV construct revealed with blue color and TLR3 displayed with red color. Red lines represent salt bridges, blue lines show Hydrogen bonds, whilst orange lines demonstrate the other contacts in docked complex. The colors of interacting residues are interpreting the characteristics of amino acids (neutral: green, Cys: yellow, aromatic: pink, aliphatic: grey, positive: blue, negative: red, and Pro&Gly: orange).

An ideal vaccine candidate must be having a strong binding potential with the immune receptor of host’s immune system to stimulate an effective and appropriate immune response. Therefore, HADDOCK v.2.2 server was utilized to conduct molecular docking analysis between our designed vaccine candidate and TLR3. TLR3 is a human immune receptor that, upon virus recognition, can applicably prompt immune responses. The docking analysis showed that the MEBV and TLR3 had strong interactions. The TLR3-MEBV binding score was 63.8 kcal/mol. The docking figures are revealed in Table 3. In the docked complex, TLR3 is showed in red, while MEBV-construct is shown in blue color respectively, as shown in (**Fig 8A**). Moreover, PDBsum was opted to achieve the conventional sketch of- interaction among the docked-complex. PDBsum is a freely available online server that gives a schematic characterization of all sorts of interactions within the docked proteins-complex. In this research work, 16 H-bond interactions were detected between MEBV and TLR3 within a range of 3.22Å (**Fig 8B**).

**Table 3:** Data of the top TLR3-MEBV docked cluster.

Parameters	Value
HADDOCK v2.2 score	-63.8 +/- 23.6
Cluster Size	4
RMSD from the overall lowest-energy structure	19.3 +/- 1.2
Van der Waals energy	-104.2 +/- 9.9
Electrostatic energy	-444.9 +/- 42.4
Desolvation energy	17.1 +/- 10.4
Restraints violation energy	2398.0+/-122.33
Buried Surface Area	3124.7+/- 82.1
Z-Score	-1.4

### *In-silico* estimation of vaccine construct

**Fig 9:**
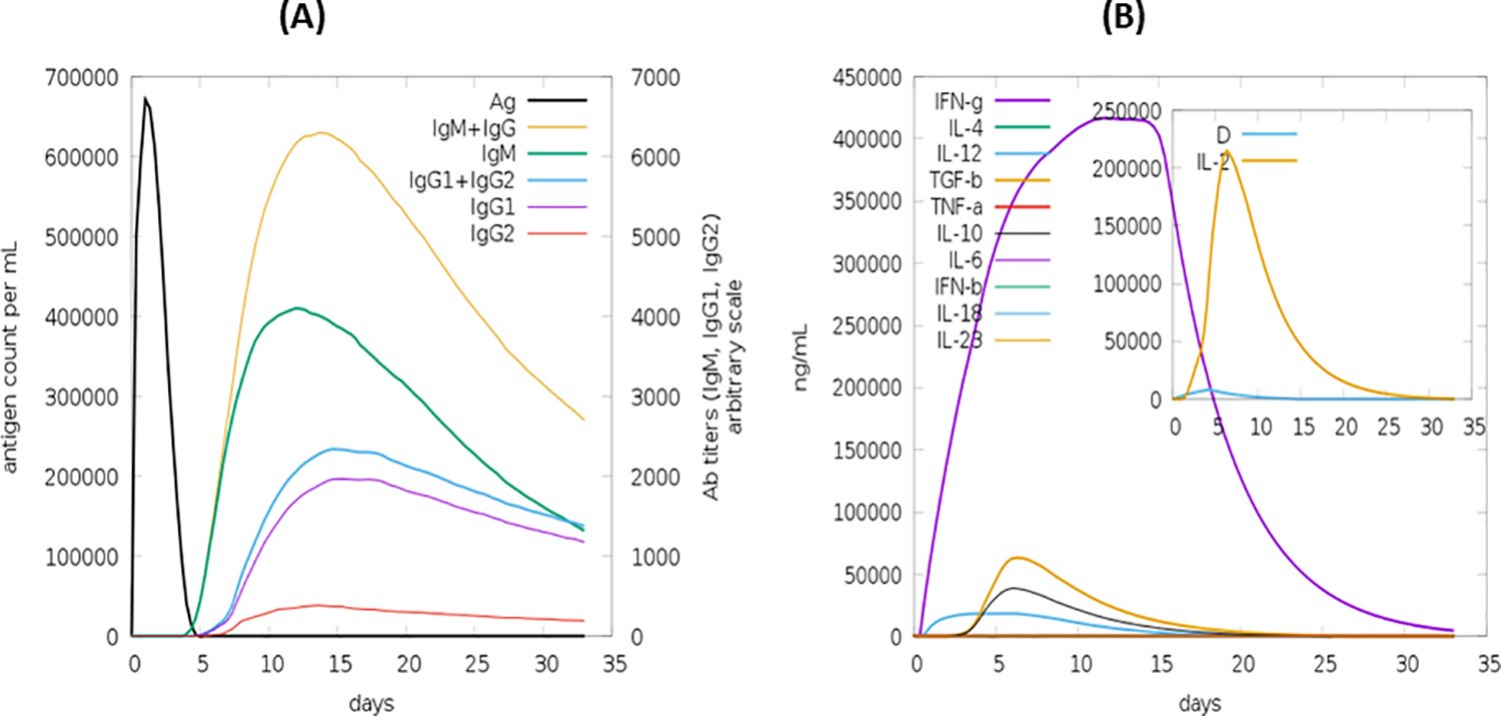
*In silico* immune response employing MEBV as an antigen. (A) Production of immunoglobulin as a result of antigen injection (B) population of B cells after three times antigen exposure.

Fig 9 displays the host immune response (*in-silico*) against our designed MEBV (act as an antigen). Among the primary responses, IgG + IgG and IgM concentration was found to be the highest one succeeded by IgM, IgG1 + IgG2 and IgG1 production at both the secondary and primary stages, accompanied by the depletion of antigen (MEBV). The *in-silico* host immune system response to the antigen is shown in **Fig 9**. Our designed MEBV also represented very strong cytokine and interleukin responses. Overall, the results of C-ImmSim 10.1 server demonstrated that the designed MEBV has a robust and appropriate immune response with clearance capability after successive encounters with pathogen.

### *In-silico* cloning

**Fig 10:**
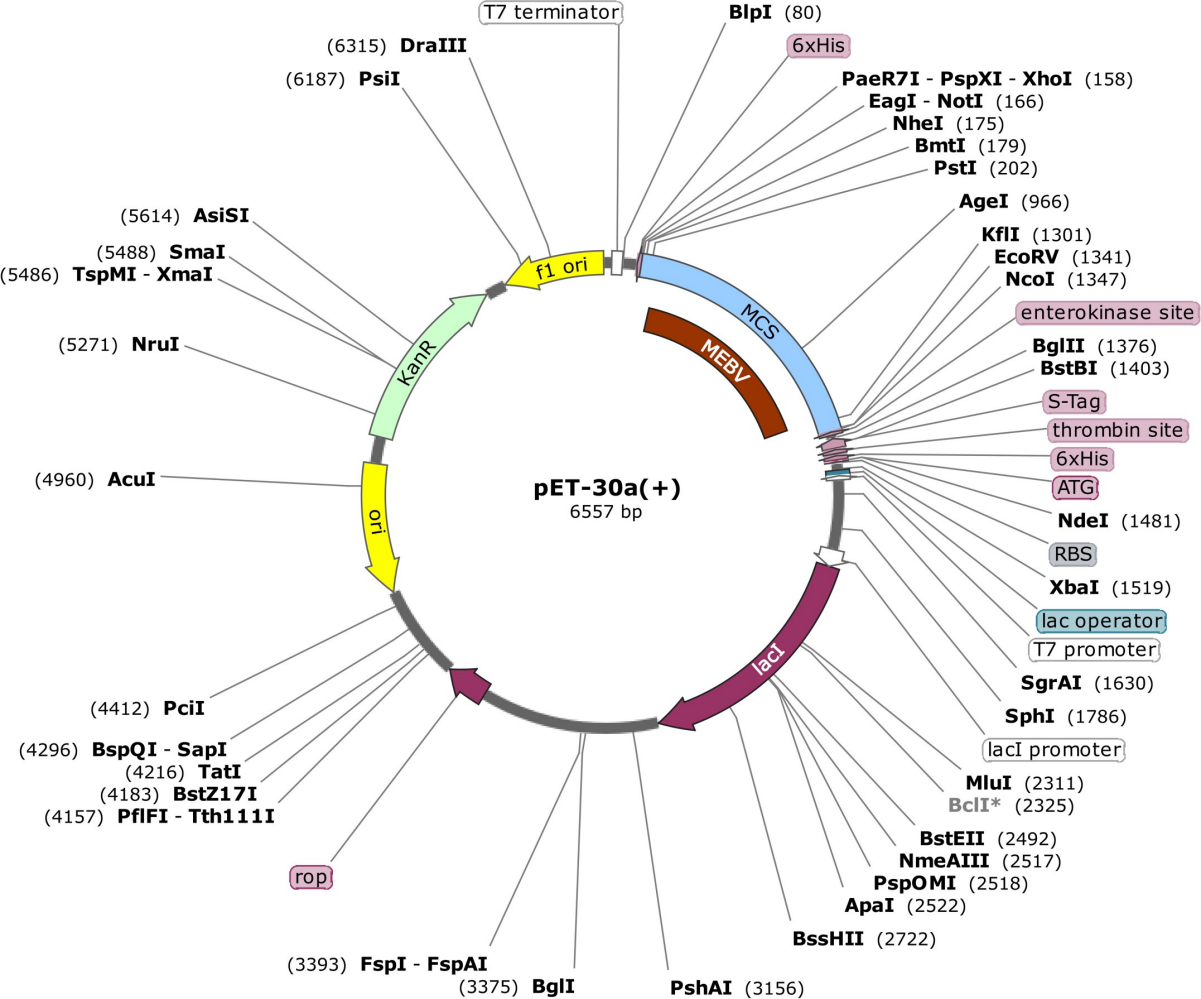
*In silico* cloning of codon optimized MEBV construct into pET28a(+) expression system. The black region exhibits the plasmid back-bone, whereas the maroon section demonstrates the inserted DNA sequence.

*In-silico* cloning was done to assure the expression of MEBV in extensively employed *E*. *coli* hosts. Before cloning, JCat server was utilized to optimize MEBV codons in accordance with *E*. *coli* (strain K12) expression system. The optimized MEBV sequence contains 1146 nucleotides, CAI value 1.0, and a GC content of 58.46%, which clearly ensuring the reliability and positive expression of our desired protein. *In-Silico* cloning was executed to validate the expression of MEBV in a host cell. This step was supported by the addition two restriction sites (BamHI and HindIII) on both ends of MEBV optimized nucleotide sequence. This modified sequence was then cloned at the multiple cloning sites of the Pet30a (+) vector (**Fig 10**). The entire clone had a size of 6557bp.

### Molecular dynamics and simulation

**Fig 11:**
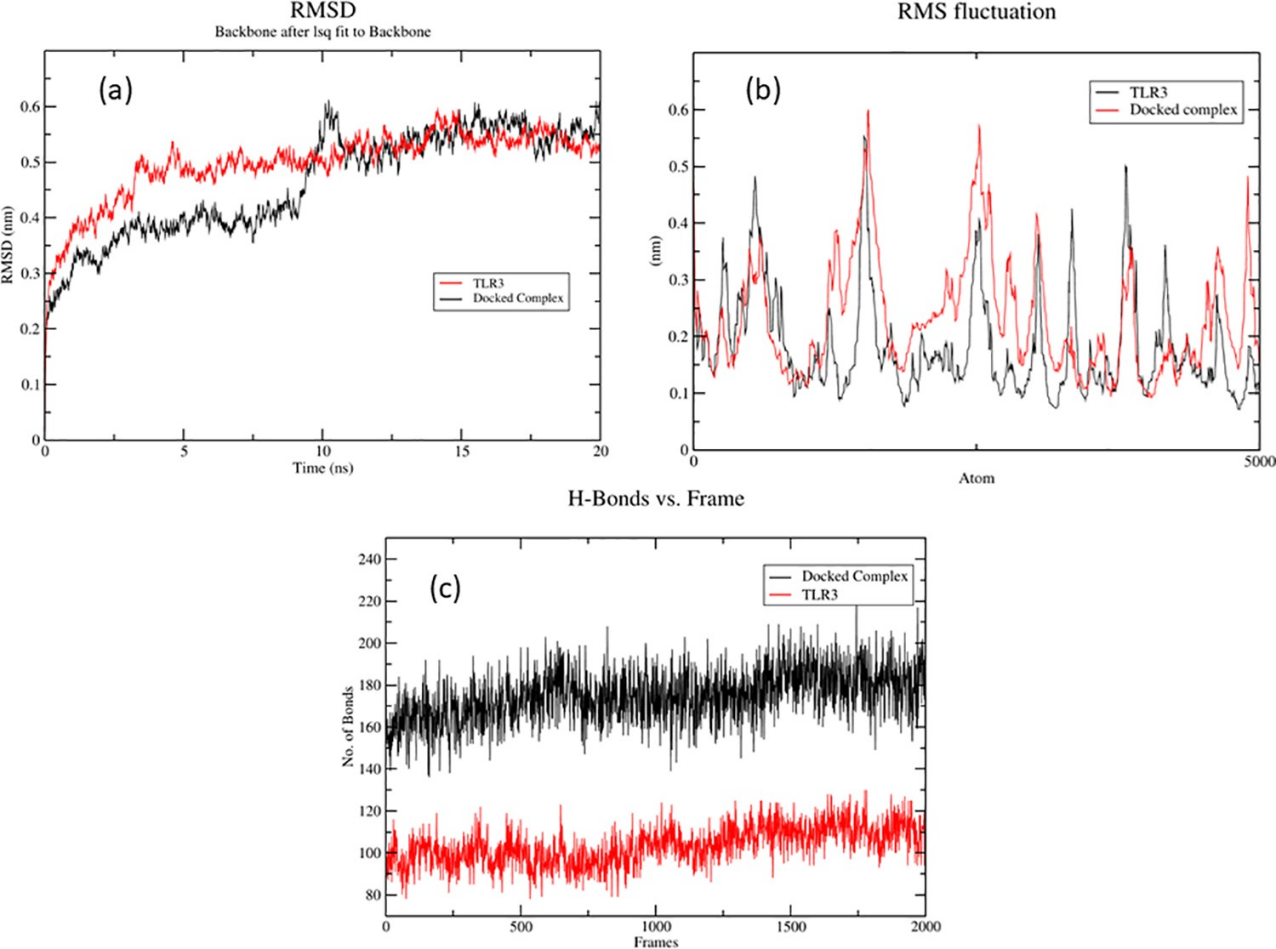
Comparisons of the RMSD, RMSF values and the number of hydrogen bonds present in the structure of MEBV-TLR3 and TLR3 protein, obtained through MD simulation. (A) RMSD of the backbone of the MEBV-TLR3 and the TLR3 Protein (B) RMSF of the backbone of the complex and the TLR3 protein (C) Hydrogen bonds present in the MEBV-TLR3 complex and the TLR3 Proteins.

In order to assess the micro interactions between the ligand and vaccine complex MD simulations are a popular method [83, 84] The structural integrity of the MEBV-TLR3 complex was determined through analysis of the Hydrogen bonds (HB), Root mean square deviations (RMSD) and Root mean square fluctuations (RMSF) carried out after performing MD simulations of 20ns. The Native structure of TLR3 not bound to any ligand, used as a control was also simulated for 20ns. The structural integrity of the MEBV-TLR3 complex was determined by calculating the RMSD of the backbone of the complex (Fig 11A) the average value of RMSD for MEBV-TLR3 was 0.4 nm and that of TLR3 was 0.45 nm. The RMSD value of MEBV-TLR3 was very stable for 10ns, after which it exhibited deviation and remained stable for the remainder of the simulation. The value of RMSD exhibited that the complex of MEBV-TLR3 endures stability for the whole 20ns of MD simulations. Additionally, RMSF figure (Fig 11B) value was calculated to estimate the residual flexibility of the backbone of MEBV-TLR3 complex. There were no major fluctuations in the structures with the average value of RMSF for MEBV-TLR3 being 0.35 nm and that of TLR3 being 0.37 nm. Hydrogen bonds present in the protein structure are the main stabilizing force. The study of hydrogen bonds in the MEBV-TLR3 complex and the TLR3 protein provide evidence that the hydrogen bond interactions taking place are stable throughout the 20ns simulation and the complex exhibits a greater number of hydrogen bonds further validating stability of the complex (Fig 11C).

## Discussion

HTLV-1 is a T-cell affecting retrovirus that usually shows no symptoms, however, some of its patients develop adult T-cell leukemia [19], HTLV-associated uveitis [21], HAM/TSP [20], or other inflammation-associated medical illnesses [5]. At present, no treatment is available for this infection, and it has severe lifelong effects; therefore, there is a need to recognize some therapeutic substances to fight against this chronic infection. Currently, vaccination is considered as the most powerful tool to boost the immune system, helping the body to combat various infectious diseases. Manufacturing and Development of an effective live or attenuated vaccine is a comparatively labour-intensive, costly and time-consuming process. Besides that, the use of traditional attenuated vaccines is also limited by certain factors, such as their weak support in boosting the immune responses and a variety of allergic reactions [85]. Multi-epitope based vaccines (MEBV) are preferred over the traditional vaccines owing to its cost-effectiveness, improved safety, and the prospect to sensibly engineer the epitopes for amplified potency [35]. MEBV also excludes all of the unwanted parts from the final vaccine construct that might lead to adverse pathological effects [86]. Currently, a variety of immunoinformatics approaches are available for the efficient designing of MEBV, Similar techniques have been used previously to suggest an appropriate MEBV for Dengue virus [87], Lassa virus [88], Hepatitis C virus [89], influenza virus [90], Respiratory Syncytial Virus [91], Zika Virus [92], Coronavirus (COVID-19) [93, 94] and many others. Even we have also followed the same methodology to propose vaccines against *Mycoplasma pneumonia* and SARS-CoV-2. Therefore, owing to extensively described worthiness and widespread adoption, immunoinformatics based methodologies were considered for the designing of an effective MEBV against pathogenic HTLV-1.

We have conducted an extensive literature review and to the best of our information, the current study is the very first research to propose an effective MEBV against HTLV-1 by exploring the whole proteome of HTLV-1. Although, Raza et al. in 2021 constructed the MEBV against the same virus, however, they only considered Tax protein to fulfill their objectives [95]. Furthermore, there study didn’t incorporate the molecular dynamic simulation studies to validate the findings of molecular docking experiments. Similarly, Alam et al. also worked on HTLV-1 virus; however, they only marked out the potential epitopes that could be employed for the development of HTLV-1 vaccine without designing complete vaccine construct. Moreover Alam et al. only employed glycoproteins to predict epitopes of interest [96], while the current study scrutinized the entire HTLV-1 proteome to screen for the efficient epitopes that could be used to construct a fully functional MEBV. Therefore, our proposed MEBV is more effectual and immunogenic against HTLV-1.

In the current study, MEBV was constructed by employing strong TCEs and BCEs, attained from three key proteins of HTLV-1 i.e. Accessory Protein p12I (AP), Envelop Glycoprotein gp 62 (GP) and Protein TAX-1 (PT). Accessory proteins of HTLV-1 play a key role in viral infectivity. They are also responsible for the harmful effects on normal function of mitochondria, alteration of genes expression and enhancement of T-lymphocyte activation [97]. Glycoproteins of HTLV-1 not only mediates the viral attachment to specific cellular receptors but also aid its entry within the cell [98]. Tax proteins of HTLV-1 are involved in the complex pathway that prompts adult T-cell leukemia [99]. Thus, all these proteins serve as suitable therapeutic targets for HTLV-1. B-lymphocytes prompts antibody production [100], HTLs provoke cellular as well as humoral immune responses [101] and CTLs are responsible for the inhibition of viral spread through the generation of antiviral cytokines. They also play an essential function in the extermination of body cells which have been infected with virus [102]. Due to high importance in provoking immune responses, both BCEs and TCEs were forecasted to design a final MEBV construct.

Ideal epitopes were chosen based on the four distinct factors, namely; antigenicity, allergenicity, immunogenicity, and toxicity. Cytokines secreting HTLs encompasses a strong potential to activate different immune cells and to overcome pro-inflammatory responses, which eventually bring down the likelihood of tissue injuries. Thus, Cytokines secreting capability of HTLs was also evaluated to ultimately pick the finest epitopes to design MEBV construct. β-defensin (an a djuvant) was firstly attached to the very first CTL through EAAAK linker while all other CTL were connected together with the aid of AAY linkers. The reason of attaching an adjuvant with the CTL was to selectively regulate both cellular and acquired immune response [103]. Additionally, Adjuvants also increases the stability of vaccine by providing defensive actions against pathogenic infections [104]. In the current research work, β-defensin was employed as adjuvant by virtue of its strong antimicrobial and immunomodulatory effects [58]. β-defensin has already been used in different published studies on MEBV construction [91, 105, 106]. Just like CTL, HTL and BCEs were also joined together via GPGPG and KK Linkers respectively. The reason of adding linkers was to increase the stabilization, folding and expression of the final developed vaccine [107]. The endmost vaccine construct was very extensive in size with a molecular weight of 42.15kDa, still, the prolonged size doesn’t hinder the expression and stability of MEBV, as supported by previous studies in which vaccines with longer sequences were proposed [88, 108, 109].

Our constructed vaccine sequence didn’t show any significant homology with human proteome, making it an ideal vaccine with no side effects on normal proteins of human body. Moreover, recombinant MEBV depicted eminent solubility against the up-regulated expression in *E*. *coli* host cells, which is another merit of this vaccine making it easily available for the host cells [110]. Results of Instability index also validated the stable nature of designed vaccine upon expression in host, consequently supplementing the usage capacity further. The theoretical pI of our proposed vaccine against HTLV-1 was found to be 9.39 which clearly represents the alkaline class of vaccine and provides a stable connection in the physiological pH range. Besides, the hydrophobic nature and thermostability of the constructed MEBV was confirmed by GRAVY score and aliphatic index respectively. Our designed vaccine showed a mean half-life of 0 h in yeast< 20 h in vivo< 30 h in vitro that is also consistent with literature [91, 111, 112].

The HLA alleles are so important because they retain the responses to TCEs, although, based on ethnicity, these alleles are extraordinary polymorphic. TCEs are expected to depict binding affinity with a lot of HLA alleles so that more population coverage can be achieved. Consequently, CTL and HTL epitopes, along with their corresponding HLA alleles, were nominated to forecast the allele distribution globally. The outcomes demonstrated that the selected epitopes and their respective alleles are found in major geographical areas of the World with global population coverage of 95.8%. The highest coverage was seen at 98% in India. The population coverage was recorded to be 97.14% and 94.06% in the United States and Japan respectively, where the discovery of HTLV-1 was made and had numerous epidemics [113].

The modeling of 3D structure provides outstanding support in the estimation of protein dynamics, function and interaction capabilities with other proteins. Therefore, 3D model of MEBV was predicted and its desirable properties were considerably increased through refinement. Ramachandran plot analysis (89.2% residues in favored region, 7.6% in allowed and only 3.2% in outer region), Errat quality factor (−4.74), Clash score (14.8), GDT-HA (0.8829), Poor Rotamers (0), MolProbity (2.250), RMSD (0.592) and Z-score (-5.53) validated the quality of final model.

For a vaccine a foremost requirement is that it must develop stable connections with immune receptors so that it can be expeditiously absorbed and transported throughout the host body [114]. Molecular docking and MD simulations were conducted to analyze the binding affinity of developed MEBV with immune receptor TLR-3. Results of these software confirmed that very small energy is required to establish a stable complex with powerful interactions between vaccine and TLR-3. These results imply that our designed MEBV can successfully bind with the immune receptors.

It is strongly needed that the designed MEBV should elicit robust cellular and humoral immune reactions. Our developed MEBV displayed elevated generation of IFN-γ with considerable activities of IL-2 and IL-10. Additionally, surplus active immunoglobulin, including IgM, IgG and there isotopes, were also reported for our MEBV. The irrelevant Simpson index also suggested a divergent immune response, which is possible only if the MEBV encompasses many BCEs and TCEs. Another important step to validate a certain vaccine construct is to perform its serological assessment [115]. Expression of the foreign genes may differ inside the genome of a host cell and the reason behind this variation is the inconsistency of mRNA codon; therefore, it is required to optimize the codon to ensure higher expression level in host cell [116]. CAI (58.46) and GC (1.0) content of optimized codon clearly indicates the upregulated expression of vaccine protein in *E*. *coli* host cell, which is the most widely employed expression system for the synthesis of recombinant protein [117, 118]. *In-silico* restriction cloning was also achieved by utilizing the pET30a (+) vector. The significance of using this vector includes the presence of S- and His-tags as the fusion partners that are important for uncomplicated protein purification. Besides that, S-tag further stabilizes the proteins with their affluence of charged and polar residues [119]. Disulphide engineering was conducted to further enhance the stability of final vaccine structure. This step remarkably augmented the thermostability of vaccine protein and also assisted in the investigation of genetic components of MEBV [120].

The current research work was carried out to suggest a vaccine construct against HTLV-1 by following the next-generation vaccine designing methodology. We are of view that our proposed vaccine will effectively generate both humoral and cell-mediated immune response. Its protein structure is not only immensely stable but also capable of interacting with immune receptors. Moreover, in immune simulation, effective immune responses were observed in real life.

Because the vaccine was designed with an adjuvant, B cell, and T cell epitopes (CTL, HTL), it can promote innate and adjuvant immunological reactions in the host body, making it an excellent and suitable candidate for HTLV-1 vaccine production. Thus, *in silico* designed MEBV vaccine against HTLV-1 can prove to be effective in stimulating specific cell mediated and humoral immune response and activating the acquired immune system against HTLV-1 in healthy individuals; treating both asymptomatic and symptomatic HTLV-1 infection; and preventing the development of further pulmonary, neurological, ophthalmological, autoimmune diseases, and rheumatoid. However, further wet lab investigations are highly needed to validate its actual potential to combat HTLV-1. However, further wet lab investigations are highly needed to validate its actual potential to combat HTLV-1.

## Conclusion

HTLV-1 infection is a global health issue that prompts ATLL and mediates many different immune-related disorders. Due to the unavailability of any therapeutic option, it is necessary to identify some preventive methods to combat this harmful viral infection. Because of the enormous advantages, Reverse vaccinology and computational techniques have been employed to construct MEBV. The current study utilized immunoinformatics and computational investigations to propose an efficacious MEBV model. We are confident that the proposed vaccine will trigger appropriate humoral and cell-mediated immune reactions. Immune simulations confirmed the immune responses of the final MEBV construct. Yet, further wet lab investigations are necessary to authorize the safety and efficiency of the designed MEBV against HTLV-1.

## Supporting information

S1 Fig 3D structures of (a) Protein TAX-1 and (b) Accessory Protein-p12I, predicted by i-TASSER and refined by GalaxyRefining2 server.
(TIF)

S2 Fig Tertiary structures of chosen epitopes estimated by PEPFOLD. (TIF)

S3 Fig Secondary structure of MEBV anticipated by PSIPRED. (TIF)

S4 Fig z-score of the MEBV construct predicted by PROSA web. (TIF)

S1 Table HTLV-1 proteins with their antigenicity value and Blastp results. (DOCX)

S2 Table Physiochemical properties of selected antigenic proteins of HTLV-1. (DOCX)

S3 Table Secondary structure details of the selected HTLV-1 proteins. (DOCX)

S4 Table Structural details of the HTLV-1 proteins predicted models. (DOCX)

S5 Table MHC-I epitopes of HTLV-1 proteins forecasted via IEDB consensus method. (DOCX)

S6 Table MHC-II epitopes of HTLV-1 proteins forecasted via IEDB consensus method. (DOCX)

S7 Table Linear B cell epitopes of HTLV-1 proteins estimated through ABCPred online tool. (DOCX)

S8 Table Conformational B cell epitopes of HTLV-1 proteins predicted by Ellipro online tool. (DOCX)

S9 Table Disulphide bonds of MEBV construct predicted by Disulphide by design v2.2. (DOCX)

S10 Table Linear B cell epitopes of MEBV anticipated via ABCPRED online tool. (DOCX)

S11 Table List of conformational epitopes of the final MEBV construct. (DOCX)

## List of abbreviations

HTLV-1: Human T-cell lymphotropic virus type 1
AP: Accessory Protein p12I
GP: Envelop Glycoprotein gp 62
PT: Protein TAX-1
MHC: Major Histocompatibility Complex
MEBV: Multiepitope-based Subunit Vaccine
