# Supplementary figures and images for: Rational design of chimeric Multiepitope Based Vaccine (MEBV) against human T-cell lymphotropic virus type 1: An integrated vaccine informatics and molecular docking based approach
Source: PLoS One. 2021 Oct 27;16(10):e0258443. doi: 10.1371/journal.pone.0258443 (PMC8550388; doi:10.1371/journal.pone.0258443)

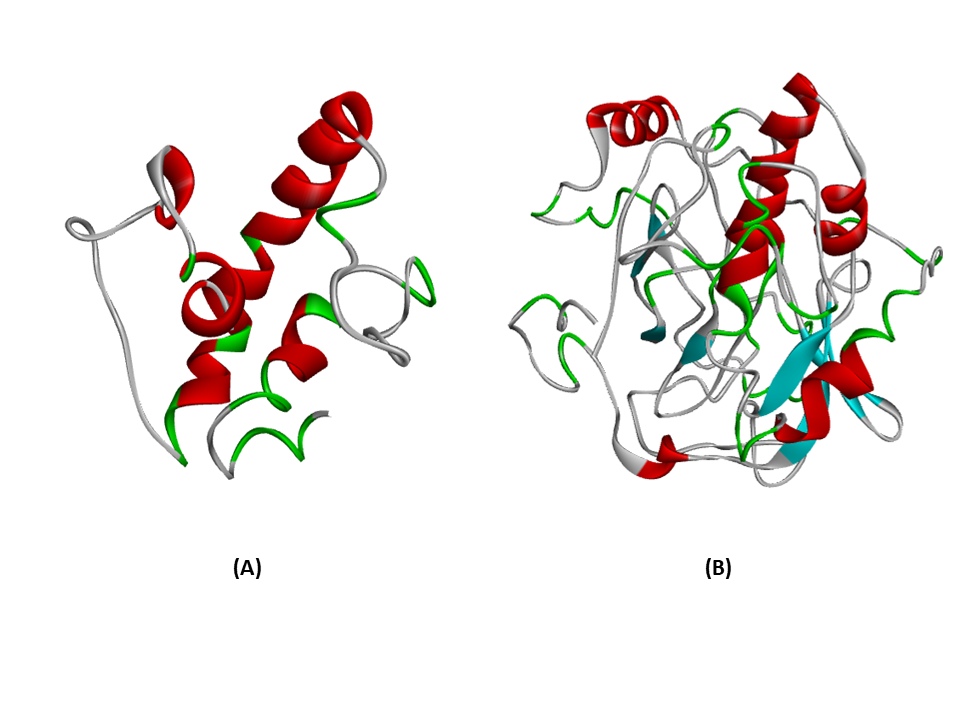

Supplement: S1 Fig — 3D structures of (a) Protein TAX-1 and (b) Accessory Protein-p12I, predicted by i-TASSER and refined by GalaxyRefining2 server. (TIF) [file pone.0258443.s001.tif]

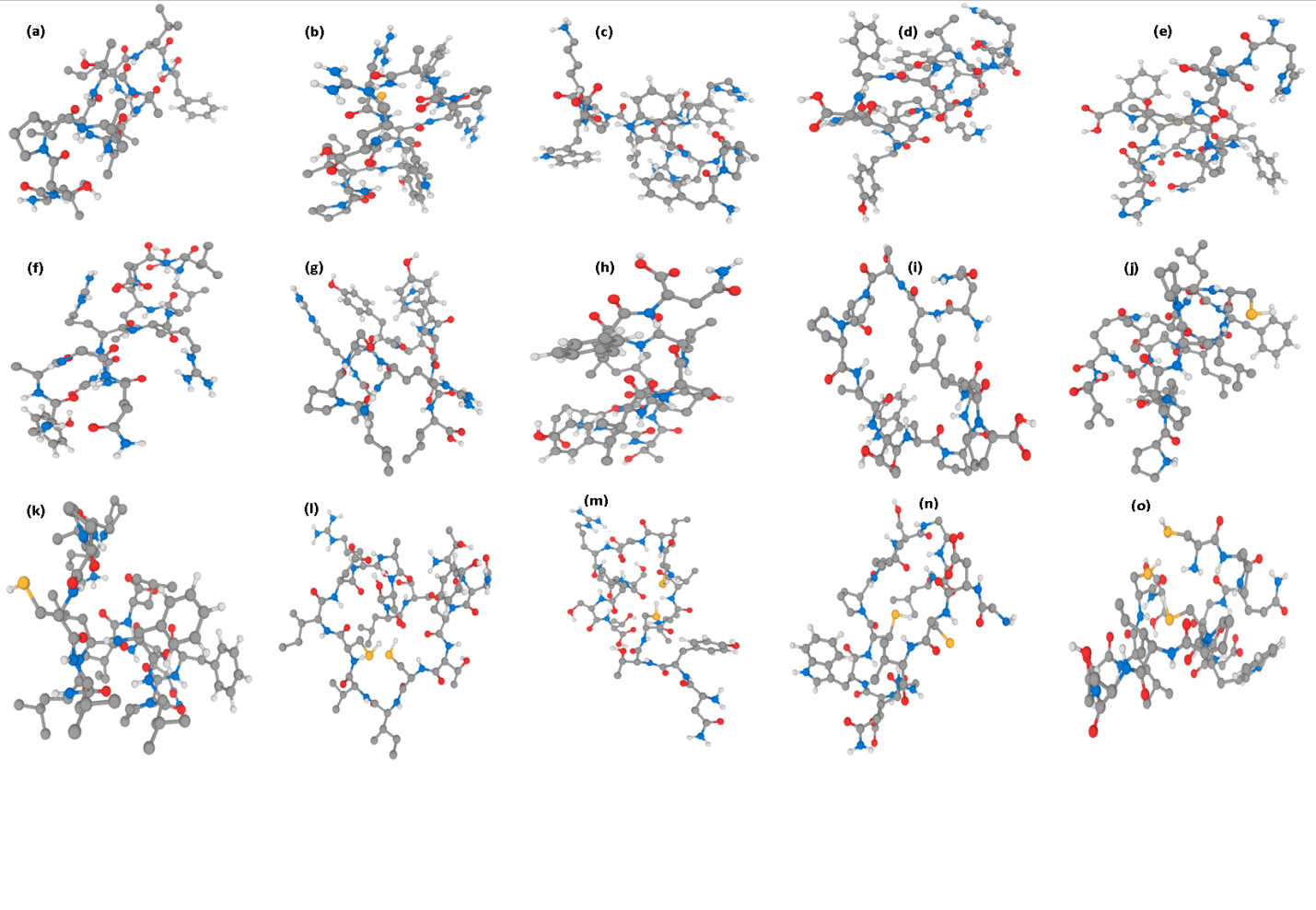

Supplement: S2 Fig — (TIF) [file pone.0258443.s002.tif]

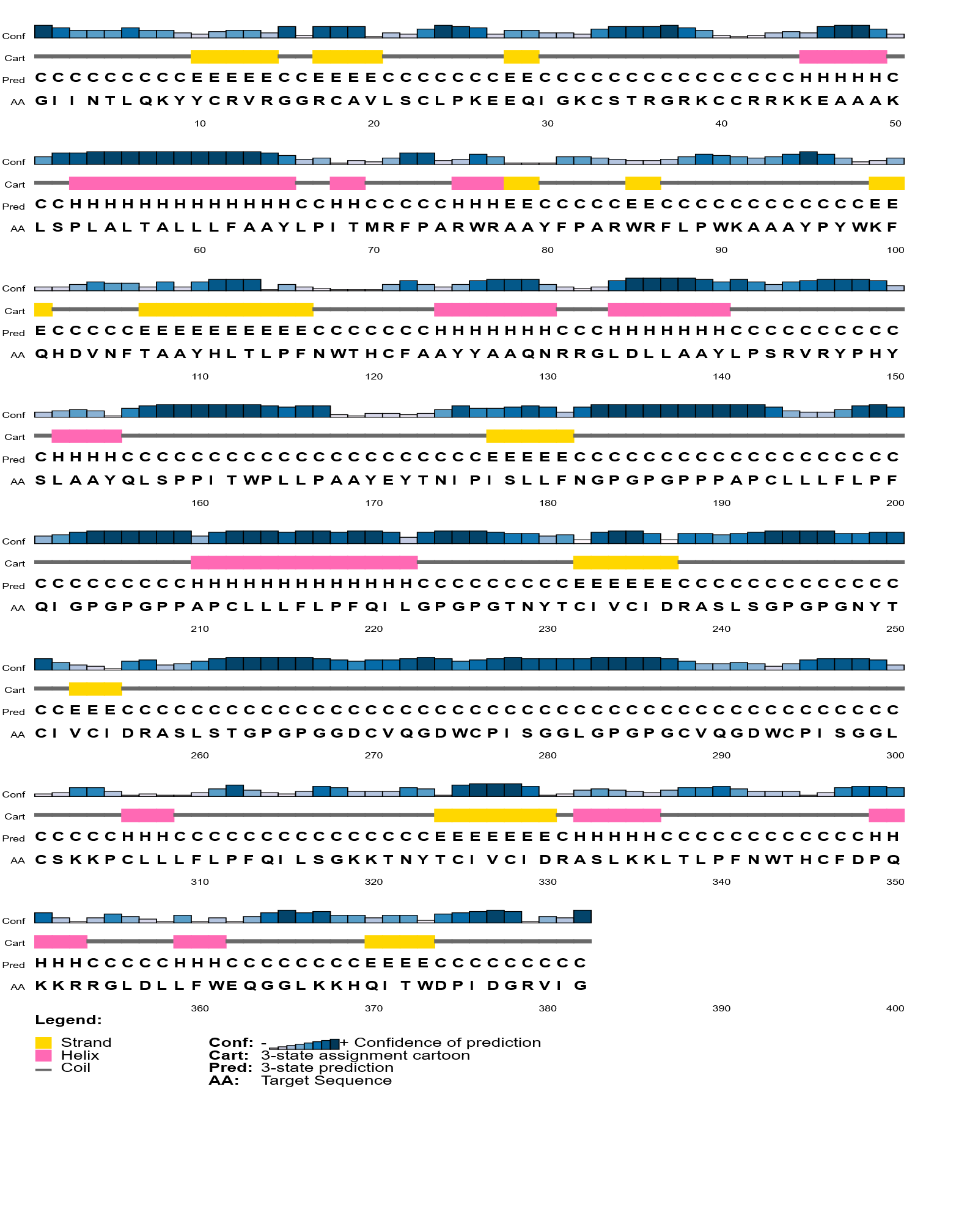

Supplement: S3 Fig — (TIF) [file pone.0258443.s003.tif]

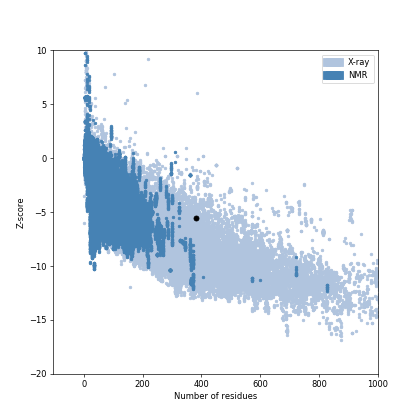

Supplement: S4 Fig — (TIF) [file pone.0258443.s004.tif]
