# Supplementary material for: Rational design of chimeric Multiepitope Based Vaccine (MEBV) against human T-cell lymphotropic virus type 1: An integrated vaccine informatics and molecular docking based approach
Source: PLoS One. 2021 Oct 27;16(10):e0258443. doi: 10.1371/journal.pone.0258443 (PMC8550388; doi:10.1371/journal.pone.0258443)
Supplement: S1 Table — (DOCX) [file pone.0258443.s005.docx]

**S1 Table:** HTLV-1 proteins with their antigenicity value and Blastp results

| **Protein** | **Accession No**  **(UniprotKB)** | **VaxiJen score**  **(threshold 0.4)** | **Blastp similarity index** |
| --- | --- | --- | --- |
| Gag-Pro Polyprotein | P10274 | 0.4358 | Max of 60% with 3% query coverage |
| Protein Rex | P03362 | 0.4511 | Max of 60% with 1% query coverage |
| Gag-Pro-Pol Polyprotein | POC205 | 0.2658 | Not determined |
| Accessory Protein p30II | POC215 | 0.3955 | Not determined |
| Accessory Protein p12I | POC214 | 0.4873 | No significant similarity found. |
| Envelop Glycoprotein gp 62 | P03381 | 0.5403 | 32.14% max similarity |
| Gag Poly Protein | P03345 | 0.4190 | Max 60% with 4% coverage |
| Basic Zipper Factor | P0C746 | 0.3453 | Not determined |
| Protein Tax 1 | P03409 | 0.4610 | No significant similarity found. |
