# Supplementary material for: Rational design of chimeric Multiepitope Based Vaccine (MEBV) against human T-cell lymphotropic virus type 1: An integrated vaccine informatics and molecular docking based approach
Source: PLoS One. 2021 Oct 27;16(10):e0258443. doi: 10.1371/journal.pone.0258443 (PMC8550388; doi:10.1371/journal.pone.0258443)
Supplement: S2 Table — (DOCX) [file pone.0258443.s006.docx]

**S2 Table:** Physiochemical properties of selected antigenic proteins of HTLV-1

| **Protein** | **Aliphatic Index** | **Molecular Weight** | **Theoretical pI** | **Half-life** | **Instability Index** | **GRAVY** |
| --- | --- | --- | --- | --- | --- | --- |
| Accessory Protein p12I | 144.95 | 11068.73 | 11.83 | 30 hours | 75.82 | 1.149 |
| Envelop Glycoprotein gp 62 | 97.52 | 53948.42 | 8.65 | 30 hours | 53.79 | 0.071 |
| Protein Tax 1 | 87.56 | 39470.53 | 6.45 | 30 hours | 48.96 | -0.054 |
