# Supplementary material for: Rational design of chimeric Multiepitope Based Vaccine (MEBV) against human T-cell lymphotropic virus type 1: An integrated vaccine informatics and molecular docking based approach
Source: PLoS One. 2021 Oct 27;16(10):e0258443. doi: 10.1371/journal.pone.0258443 (PMC8550388; doi:10.1371/journal.pone.0258443)
Supplement: S3 Table — (DOCX) [file pone.0258443.s007.docx]

**S3 Table:** Secondary structure details of the selected HTLV-1 proteins

| Proteins | α-helix | β-sheet | Coils |
| --- | --- | --- | --- |
| Accessory Protein p12I | 29.29% | 23.23% | 47.47% |
| Envelop Glycoprotein gp 62 | 23.77% | 8.61% | 47.13% |
| Protein Tax 1 | 16.43% | 15.86% | 67.71% |
