# Supplementary material for: Rational design of chimeric Multiepitope Based Vaccine (MEBV) against human T-cell lymphotropic virus type 1: An integrated vaccine informatics and molecular docking based approach
Source: PLoS One. 2021 Oct 27;16(10):e0258443. doi: 10.1371/journal.pone.0258443 (PMC8550388; doi:10.1371/journal.pone.0258443)
Supplement: S4 Table — (DOCX) [file pone.0258443.s008.docx]

**S4_Table:** Structural details of the HTLV-1 proteins predicted models

| Protein | Tools used | C-Score | Z-score | Errat value | Ramachandran Plot | | |
| --- | --- | --- | --- | --- | --- | --- | --- |
|  |  |  |  |  | Fav region | Allowed region | Disallowed region |
| Accessory Protein p12I | I-Tasser | 3.8 | -4.87 | 64.0449 | 87.6% | 8.2% | 4.1% |
| Protein Tax 1 | I-Tasser | 4.79 | -5.36 | 51.0145 | 78.3% | 13.7% | 8.0% |
