# Supplementary material for: Rational design of chimeric Multiepitope Based Vaccine (MEBV) against human T-cell lymphotropic virus type 1: An integrated vaccine informatics and molecular docking based approach
Source: PLoS One. 2021 Oct 27;16(10):e0258443. doi: 10.1371/journal.pone.0258443 (PMC8550388; doi:10.1371/journal.pone.0258443)
Supplement: S5 Table — (DOCX) [file pone.0258443.s009.docx]

S5 Table: MHC class I epitopes of HTLV-1 proteins predicted by IEDB consensus method

| Protein | Epitopes | Position | Alleles | Antigenicity |
| --- | --- | --- | --- | --- |
| Accessory Protein p12I | SLPITMRFPARW | 71-82 | HLA-B*57:01,HLA-B*53:01,HLA-B*58:01 | 0.9073 |
|  | FPARWRFLPWKA | 78-89 | HLA-B*07:02 ,HLA-B*27:05,HLA-B*53:01 | 1.4942 |
|  | LPITMRFPARWR | 72-83 | HLA-A*31:01 ,HLA-B*53:01,HLA-B*58:01, HLA-B*57:01 | 1.1198 |
|  | FLPLFFSLPLLL | 57-68 | HLA-B*35:03, HLA-A*02:01,HLA-B*53:01,HLA-E*01:01,HLA-B*51:01 | 0.6892 |
|  | LLFLPLFFSLPL | 55-66 | HLA-B*35:03,HLA-A*02:01,HLA-B*53:01 ,HLA-B*51:01 | 0.5547 |
|  | LPLFFSLPLLLS | 58-69 | HLA-B*51:01,HLA-B*35:03,HLA-B*53:01 | 0.7014 |
|  | LSPLALTALLLF | 9-20 | HLA-B*58:01,HLA-B*57:01 | 1.0379 |
| Envelop Glycoprotein gp 62 | LPAPHLTLPFNW | 262-283 | HLA-B*53:01,HLA-B*35:01,HLA-B*07:02,HLA-B*35:03 | 0.8406 |
|  | FPNITNSHVPIL | 402-413 | HLA-B*35:03,HLA-B*51:01,  HLA-B*53:01,HLA-B*35:01 | 0.5912 |
|  | RFPNITNSHVPI | 401-412 | HLA-B*35:03,HLA-B*51:01 ,HLA-B*53:01 | 0.6486 |
|  | WAREALQTGITL | 438-449 | HLA-B*40:01,HLA-B*40:02 | 0.5946 |
|  | FFQFCPLIFGDY | 11-22 | HLA-A*29:02,HLA-B*15:02,HLA-A*30:02 | 0.8020 |
|  | LPSRVRYPHYSL | 470-481 | HLA-B*07:02,HLA-B*08:01,HLA-B*38:01,HLA-C*07:02 | 1.0175 |
|  | YSLYLFPHWTKK | 80-91 | HLA-A*03:01,HLA-A*11:01,HLA-B*58:01 | 0.8054 |
|  | VSRLNINLHFSK | 145-156 | HLA-A*11:01,HLA-A*03:01 | 0.6136 |
|  | AREALQTGITLV | 439-450 | HLA-B*40:02,HLA-B*40:01 | 0.7226 |
|  | ATYSLYLFPHWT | 78-89 | HLA-B*58:01,HLA-A*23:01,HLA-A*24:02 | 0.6849 |
|  | REALQTGITLVA | 440-451 | HLA-B*40:02 ,HLA-B*40:02 | 0.6492 |
|  | LILFFQFCPLIF | 8-19 | HLA-A*23:01,HLA-A*29:02,HLA-B*15:01 | 0.7215 |
|  | QEVSRLNINLHF | 143-154 | HLA-B*27:05,HLA-B*44:03,HLA-B*44:02 | 0.9402 |
|  | YAAQNRRGLDLL | 374-385 | HLA-C*06:02,HLA-C*03:03 | 1.0626 |
|  | PYWKFQHDVNFT | 131-142 | HLA-C*07:02,HLA-A*23:01 | 1.1361 |
|  | LTLPFNWTHCFD | 267-278 | HLA-B*57:01,HLA-B*35:01,HLA-A*32:01 | 0.8995 |
|  | SPYWKFQHDVNF | 130-141 | HLA-A*23:01, HLA-C*07:02 | 0.7911 |
|  | YWKFQHDVNFTQ | 132-143 | HLA-A*23:01,HLA-C*07:02 | 0.8964 |
|  | SHVPILQERPPL | 408-419 | HLA-B*35:03,HLA-B*38:01 | 0.7495 |
|  | HLTLPFNWTHCF | 266-277 | HLA-A*32:01,HLA-B*57:01,HLA-A*32:01,HLA-B*15:02 | 1.0691 |
|  | WTHCFDPQIQAI | 273-284 | HLA-C*08:02,HLA-B*38:01 | 0.5413 |
|  | THCFDPQIQAIV | 275-286 | HLA-C*08:02,HLA-B*38:01 | 0.8572 |
| Protein Tax 1 | FQPARAPVTLTA | 218-229 | HLA-E*01:01,HLA-B*39:01  HLA-B*35:03,HLA-A*02:06 | 0.6942 |
|  | LSPPITWPLLPH | 159-170 | HLA-E*01:01, HLA-B*35:03 | 0.5060 |
|  | QLSPPITWPLLP | 158-169 | HLA-E*01:01 ,HLA-B*35:03 | 0.7867 |
|  | FEEYTNIPISLL | 309-320 | HLA-B*40:01,HLA-E*01:01 | 0.6489 |
|  | YQLSPPITWPLL | 157-168 | HLA-E*01:01,HLA-B*39:01 HLA-B*48:01,HLA-A*02:06,HLA-B*38:01,HLA-C*07:02 | 0.6726 |
|  | LFEEYTNIPISL | 308-319 | HLA-B*40:01,HLA-C*07:02 | 0.6092 |
|  | LPFHSTLTTPGL | 235-246 | HLA-B*39:01,HLA-B*51:01  HLA-B*07:02 | 0.5581 |
|  | IFCHPGQLGAFL | 172-183 | HLA-C*03:03,HLA-B*38:01,HLA-B*35:03 | 0.5873 |
|  | HLLFEEYTNIPI | 306-317 | HLA-B*40:01 HLA-A*02:01 | 0.5957 |
|  | EYTNIPISLLFN | 311-322 | HLA-A*24:02,HLA-A*23:01  HLA-A*29:02 | 1.0096 |
|  | GQLGAFLTNVPY | 177-188 | HLA-B*15:01,sHLA-B*15:02 | 0.5524 |
