# Supplementary material for: Rational design of chimeric Multiepitope Based Vaccine (MEBV) against human T-cell lymphotropic virus type 1: An integrated vaccine informatics and molecular docking based approach
Source: PLoS One. 2021 Oct 27;16(10):e0258443. doi: 10.1371/journal.pone.0258443 (PMC8550388; doi:10.1371/journal.pone.0258443)
Supplement: S6 Table — (DOCX) [file pone.0258443.s010.docx]

S6 Table: MHC class II epitopes of HTLV-1 proteins predicted by IEDB consensus method

| **Protein** | **Epitopes** | **Position** | **Alleles** | **Antigenicity** |
| --- | --- | --- | --- | --- |
| Accessory Protein p12I | FFSLPLLLSPSLPIT | 61-75 | HLA-DRB1*07:03,HLA-DRB1*13:02,HLA-DRB1*07:01,HLA-DRB1*11:28, HLA-DRB1*13:05,HLA-DRB1*15:01,HLA-DRB1*04:04,HLA-DRB1*13:07,HLA-DRB1*01:01 | 1 0.6063 |
|  | FSLPLLLSPSLPITM | 62-76 | HLA-DRB1*07:03,HLA-DRB1*13:02 ,HLA-DRB1*07:01 HLA-DRB1*15:01,HLA-DRB1*04:04 | 0.6063 |
|  | LPLLLSPSLPITMRF | 64-78 | HLA-DRB1*07:03, HLA-DRB1*13:02,HLA-DRB1*07:01 HLA-DRB1*15:01 | 0.9041 |
|  | PLLLSPSLPITMRFP | 65-79 | HLA-DRB1*07:03,HLA-DRB1*07:01,HLA-DRB1*13:02 | 0.6864 |
|  | SLPLLLSPSLPITMR | 63-77 | HLA-DRB1*07:03,HLA-DRB1*13:02 ,HLA-DRB1*07:01 HLA-DRB1*15:01 | 0.9150 |
|  | FRLLSPLSPLALTAL | 3-17 | HLA-DRB1*11:28,HLA-DRB1*13:05 0.2,HLA-DRB1*13:21 HLA-DRB1*13:07,HLA-DRB1*09:01,HLA-DRB1*01:02  HLA-DRB1*11:20,HLA-DRB1*01:01, HLA-DRB1*04:21 HLA-DRB1*11:04, HLA-DRB1*11:06,HLA-DRB1*13:11 | 0.8284 |
|  | FLPFQILSGLLFLLF | 43-57 | HLA-DRB1*11:28,HLA-DRB1*13:05,HLA-DRB1*01:02  HLA-DRB1*15:02,HLA-DRB5*01:05,HLA-DRB5*01:01,  HLA-DRB1*13:21,HLA-DRB1*11:01 | 0.5074 |
|  | LLFLPFQILSGLLFL | 41-55 | HLA-DRB1*11:28,HLA-DRB1*13:05,HLA-DRB1*01:02 0.73, HLA-DRB1*15:02,HLA-DRB5*01:05,HLA-DRB1*13:21,HLA-DRB1*11:01 | 0.5763 |
|  | APCLLLFLPFQILSG | 37-51 | HLA-DRB1*15:06,HLA-DRB1*15:01,HLA-DRB1*15:02,HLA-DRB1*01:02,HLA-DRB1*11:28,HLA-DRB1*13:05 | 0.5904 |
|  | LPLFFSLPLLLSPSL | 58-72 | HLA-DRB1*15:06,HLA-DRB1*11:28,HLA-DRB1*13:05 HLA-DRB1*07:03, HLA-DRB1*15:02, HLA-DRB1*04:01,  HLA-DRB1*13:07 | 0.6257 |
|  | MLFRLLSPLSPLALT | 1-15 | HLA-DRB1*11:28,HLA-DRB1*13:05, HLA-DRB1*04:01, HLA-DRB1*13:21, HLA-DRB1*09:01,HLA-DRB1*13:07  HLA-DRB1*04:04, HLA-DRB1*01:02, HLA-DRB1*01:01, HLA-DRB1*12:01,  HLA-DRB1*11:20, HLA-DRB1*04:05, HLA-DRB1*04:21, HLA-DRB1*11:04, HLA-DRB1*11:06, HLA-DRB1*13:11 | 0.5898 |
|  | LLFLPLFFSLPLLLS | 55-69 | HLA-DRB1*15:06, HLA-DRB1*11:28, HLA-DRB1*13:05, HLA-DRB1*15:01, HLA-DRB1*07:03, HLA-DRB1*15:02,  HLA-DRB1*13:07,HLA-DRB1*04:01 | 0.6460 |
|  | PCLLLFLPFQILSGL | 38-52 | HLA-DRB1*15:06,HLA-DRB1*15:01, HLA-DRB1*15:02, HLA-DRB1*01:02, HLA-DRB1*11:28, HLA-DRB1*13:05 | 0.6247 |
|  | PAPCLLLFLPFQILS | 36-50 | HLA-DRB1*15:06,HLA-DRB1*15:01, HLA-DRB1*15:02,  HLA-DRB1*01:02 | 0.6164 |
|  | FLPLFFSLPLLLSPS | 57-71 | HLA-DRB1*15:06,HLA-DRB1*11:28,HLA-DRB1*13:05, HLA-DRB1*07:03,HLA-DRB1*04:01, HLA-DRB1*15:02, HLA-DRB1*13:07 | 0.6806 |
|  | PPPAPCLLLFLPFQI | 34-48 | HLA-DRB1*15:06, HLA-DRB1*15:01, HLA-DRB1*15:02 HLA-DRB1*01:02 | 0.7086 |
|  | PPAPCLLLFLPFQIL | 35-49 | HLA-DRB1*15:06, HLA-DRB1*15:01, HLA-DRB1*15:02, HLA-DRB1*01:02 | 0.6695 |
|  | PLFFSLPLLLSPSLP | 59-73 | HLA-DRB1*11:28,HLA-DRB1*13:05, HLA-DRB1*07:03  HLA-DRB1*04:01, HLA-DRB1*13:07 | 0.5609 |
| Envelop Glycoprotein gp 62 | TNYTCIVCIDRASLS | 221-235 | HLA-DRB1*03:06,HLA-DRB1*03:07, HLA-DRB1*03:08,HLA-DRB1*11:07, HLA-DRB5*01:05 | 1.0023 |
|  | NYTCIVCIDRASLST | 222-236 | HLA-DRB1*03:06,HLA-DRB1*03:07,  HLA-DRB1*03:08, HLA-DRB1*11:07, HLA-DRB5*01:05 | 0.9098 |
|  | CIVCIDRASLSTWHV | 225-239 | HLA-DRB1*03:06,HLA-DRB1*03:07, HLA-DRB1*03:08,HLA-DRB1*11:07 | 0.5254 |
|  | LQTGITLVALLLLVI | 453-467 | HLA-DRB1*11:28,HLA-DRB1*13:05, HLA-DRB1*15:06,HLA-DRB1*11:04, HLA-DRB1*11:06,HLA-DRB1*13:11 | 0.8425 |
|  | QTGITLVALLLLVILY | 444-458 | HLA-DRB1*11:28,HLA-DRB1*13:05,  HLA-DRB1*15:06,HLA-DRB1*11:04, HLA-DRB1*11:06,HLA-DRB1*13:11 | 0.8225 |
|  | TGITLVALLLLVILA | 445-459 | HLA-DRB1*11:28,HLA-DRB1*13:05, HLA-DRB1*15:06, HLA-DRB1*11:04,HLA-DRB1*11:06,  HLA-DRB1*13:11 | 0.8384 |
|  | GITLVALLLLVILAG | 446-460 | HLA-DRB1*11:04,HLA-DRB1*11:06  HLA-DRB1*13:11,HLA-DRB1*11:28 HLA-DRB1*13:05,HLA-DRB1*13:21 HLA-DRB1*15:06 | 0.6872 |
|  | ITLVALLLLVILAGP | 209-223 | HLA-DRB1*13:21,HLA-DRB1*15:06 HLA-DRB1*11:28,HLA-DRB1*13:05  HLA-DRB1*11:04,HLA-DRB1*11:06 HLA-DRB1*13:11 | 0.5283 |
|  | QLRHLPSRVRYPHYS |  | HLA-DRB5*01:01,HLA-DRB5*01:05 | 0.6510 |
|  | SYHATYSLYLFPHWT | 75-89 | HLA-DRB1*07:03,HLA-DRB1*07:01 | 0.7921 |
|  | SSYHATYSLYLFPHW | 74-88 | HLA-DRB1*07:03,HLA-DRB1*07:01 | 0.6785 |
| Protein Tax 1 | LLYKISLTTGALIIL | 194-208 | HLA-DRB1*07:01,HLA-DRB1*04:26, HLA-DRB1*04:21,HLA-DRB1*04:08, HLA-DRB1*09:01,HLA-DRB1*03:05 | 0.5132 |
|  | VQGDWCPISGGLCSA | 24-38 | HLA-DRB1*11:07, HLA-DRB1*03:05, HLA-DRB1*03:09 | 0.9605 |
|  | YSSFHSLHLLFEEYT | 299-313 | HLA-DRB1*13:21,HLA-DRB1*15:02, HLA-DRB1*07:01 | 0.7089 |
|  | CVQGDWCPISGGLCS | 23-37 | HLA-DRB1*11:07,HLA-DRB1*03:05, HLA-DRB1*03:09 | 1.1086 |
|  | GDCVQGDWCPISGGL | 21-35 | HLA-DRB1*11:07,HLA-DRB1*03:05, HLA-DRB1*03:09 | 1.1352 |
|  | VFGDCVQGDWCPISG | 19-33 | HLA-DRB1*11:07,HLA-DRB1*03:05, HLA-DRB1*03:09 | 0.5899 |
|  | YVFGDCVQGDWCPIS | 18-32 | HLA-DRB1*11:07,HLA-DRB1*03:05 HLA-DRB1*03:09 | 0.6011 |
|  | PLLPHVIFCHPGQLG | 166-180 | HLA-DRB1*13:04,HLA-DRB1*11:02,  HLA-DRB1*11:21,HLA-DRB1*13:22 | 0.6907 |
|  | LLPHVIFCHPGQLGA | 167-181 | HLA-DRB1*13:04,HLA-DRB1*11:02,  HLA-DRB1*11:21,HLA-DRB1*13:22 | 0.5626 |
|  | CMYLYQLSPPITWPL | 153-167 | HLA-DRB1*01:01 HLA-DRB1*04:05 | 0.5246 |
