# Supplementary material for: Rational design of chimeric Multiepitope Based Vaccine (MEBV) against human T-cell lymphotropic virus type 1: An integrated vaccine informatics and molecular docking based approach
Source: PLoS One. 2021 Oct 27;16(10):e0258443. doi: 10.1371/journal.pone.0258443 (PMC8550388; doi:10.1371/journal.pone.0258443)
Supplement: S7 Table — (DOCX) [file pone.0258443.s011.docx]

S7 Table: Linear B cell Epitopes of HTLV-1 proteins predicted by ABCPred server

| **Protein** | **Epitopes** | **Position** | **Antigenicity** |
| --- | --- | --- | --- |
| Accessory Protein p12I | PCLLLFLPFQILSG | 38 | 0.6293 |
| Envelop Glycoprotein gp 62 | RRGLDLLFWEQGGL | 379 | 0.9600 |
|  | YAAQNRRGLDLLFW | 374 | 0.8787 |
|  | LFFQFCPLIFGDYS | 10 | 0.9151 |
|  | SYHATYSLYLFPHW | 75 | 0.7372 |
|  | LALPAPHLTLPFNW | 260 | 0.7689 |
|  | QEQCRFPNITNSHV | 397 | 0.7374 |
|  | LATLILFFQFCPLI | 260 | 0.5975 |
|  | TNYTCIVCIDRASL | 221 | 1.0062 |
|  | LTLPFNWTHCFDPQ | 267 | 1.2029 |
|  | NITNSHVPILQERP | 404 | 0.8678 |
|  | AMGAGVAGGITGSM | 325 | 0.5917 |
|  | YDPIWFLNTEPSQL | 170 | 0.9479 |
| Protein Tax 1 | LATCPEHQITWDPI | 46 | 0.5766 |
|  | HQITWDPIDGRVIG | 52 | 0.8070 |
|  | IFCHPGQLGAFLTN | 172 | 0.5385 |
|  | GLLPFHSTLTTPGL | 233 | 0.6914 |
