# Supplementary material for: Rational design of chimeric Multiepitope Based Vaccine (MEBV) against human T-cell lymphotropic virus type 1: An integrated vaccine informatics and molecular docking based approach
Source: PLoS One. 2021 Oct 27;16(10):e0258443. doi: 10.1371/journal.pone.0258443 (PMC8550388; doi:10.1371/journal.pone.0258443)
Supplement: S8 Table — (DOCX) [file pone.0258443.s012.docx]

S8 Table: Conformational B cell Epitopes of proteins predicted by Ellipro server

| Protein | **Residues** | **No. of Residues** | **Score** |
| --- | --- | --- | --- |
| Accessory Protein p12I | A:Q298, A:S300, A:S301, A:F302, A:H303 | 5 | 0.752 |
|  | A:P5, A:G6, A:F7, A:G8, A:Q9, A:S10, A:L11, A:L12, A:F13, A:G14, A:Y15, A:P16, A:V17, A:Y18, A:V19, A:F20, A:G21, A:D22, A:C23, A:V24, A:Q25, A:G26, A:D27, A:W28, A:C29, A:P30, A:I31, A:S32, A:G33, A:G34, A:L35, A:C36, A:S37, A:A38, A:R39, A:L40, A:P50, A:E51, A:Q53, A:T55, A:W56, A:D57, A:P58, A:I59, A:D60, A:G61, A:R62, A:V63, A:G65, A:S66, A:Q69, A:F70, A:P73, A:L75, A:P76, A:S77, A:F78, A:P79, A:T80, A:Q81, A:R82, A:T83, A:S84, A:K85, A:T86, A:L87, A:K88, A:V89, A:L90, A:T91, A:P92, A:P93, A:I94, A:T95, A:H96, A:T97, A:T98, A:P99, A:N100, A:I101, A:P102, A:P103, A:S104, A:F105, A:L106, A:Q107, A:A108, A:R110, A:K111, A:Y112, A:F115, A:N117, A:G118, A:Y119, A:M120, A:E121, A:P122, A:T123, A:L124, A:G125, A:Q126, A:H127, A:L128, A:P129, A:T130, A:H175, A:G177, A:A181, A:L183, A:T184, A:N185, A:V186, A:P187, A:Y188, A:K189, A:R190, A:I191, A:E192, A:E193, A:H279, A:K280, A:F281, A:Q282, A:T283, A:K284, A:A285, A:Y286, A:H287, A:P288, A:L291, A:E311, A:T313 | 132 | 0.659 |
|  | A:M1, A:A2, A:H3, A:F4, A:S132, A:F133, A:P134, A:D135, A:P136, A:G137, A:L138, A:R139, A:P140, A:Q141, A:N142, A:L143, A:Y144, A:T145, A:L146, A:W147, A:G148, A:G149, A:S150, A:V151, A:V152, A:C153, A:M154, A:Y155, A:L156, A:Y157, A:Q158, A:L159, A:S160, A:P161, A:P162, A:I163, A:T164, A:P209, A:E210, A:D211, A:C212, A:L213, A:Q231, A:D252, A:G253, A:T254, A:P255, A:M256, A:I257, A:S258, A:G259, A:P260, A:C261, A:P262, A:K263, A:D264, A:G265, A:Q266, A:P267, A:S268, A:L269, A:V270, A:H331, A:E332, A:P333, A:Q334, A:I335, A:S336, A:P337, A:G338, A:G339, A:L340, A:E341, A:P342, A:P343, A:S344, A:E345, A:K346, A:H347, A:F348, A:R349, A:E350, A:T351, A:E352, A:V353 | 85 | 0.602 |
| Envelop Glycoprotein gp 62 | A:A370, A:A371, A:M372, A:S373, A:L374, A:A375, A:S376, A:G377, A:K378, A:S379, A:L380, A:L381, A:H382, A:E383, A:V384, A:D385, A:K386, A:D387, A:I388, A:S389, A:Q390, A:L391, A:T392, A:Q393, A:A394, A:I395, A:V396, A:K397, A:N398, A:H399, A:K400, A:N401, A:L402, A:L403, A:K404, A:I405, A:A406, A:Q407, A:Y408, A:A409, A:A410, A:Q411, A:N412, A:R413, A:R414, A:G415, A:L416, A:D417, A:L418, A:L419, A:F420, A:W421, A:E422, A:Q423, A:G424, A:G425, A:L426, A:C427, A:K428, A:A429, A:L430, A:Q431, A:E432, A:Q433, A:C434, A:C435, A:F436, A:L437, A:N438, A:I439, A:T440, A:N441, A:S442, A:H443, A:V444, A:S445, A:I446, A:L447, A:Q448, A:E449, A:R450, A:P451, A:P452, A:L453, A:E454, A:N455 | 86 | 0.856 |
|  | A:G6, A:K7, A:L8, A:A22, A:E23, A:V24, A:G25, A:K26, A:K27, A:F28, A:E29, A:K30, A:D31, A:T32, A:G33, A:I34, A:K35, A:V36, A:G55, A:D59, A:L77, A:A78, A:E79, A:I80, A:T81, A:P82, A:D83, A:K84, A:A85, A:F86, A:Q87, A:D88, A:K89, A:L90, A:Y91, A:R99, A:Y100, A:N101, A:G102, A:K103, A:L104, A:I105, A:N268, A:A269, A:A270, A:S271, A:P272, A:N273, A:K274, A:E275, A:L276, A:A277, A:K278, A:E279, A:F280, A:E282, A:N283, A:Y284, A:L285, A:L286, A:T287, A:D288, A:E289, A:G290, A:E292, A:A293, A:K296, A:L305, A:K306, A:S307, A:E310 | 71 | 0.637 |
|  | A:Y118, A:N119, A:K120, A:D121, A:L122, A:L123, A:P124, A:N125, A:P126, A:P127, A:K128, A:T129, A:E131, A:E132, A:I133, A:P134, A:A135, A:L136, A:D137, A:K138, A:E139, A:L140, A:K141, A:A142, A:K143, A:G144, A:K145, A:S146, A:A147, A:L148, A:M149, A:D165, A:G166, A:Y168, A:K171, A:Y172, A:E173, A:N174, A:G175, A:K176, A:Y177, A:D178, A:I179, A:K180, A:D181, A:V182, A:G183, A:V184, A:D185, A:N186, A:A187, A:G188, A:K190, A:A191, A:T194, A:F195, A:D198, A:L199, A:K201, A:N202, A:K203, A:H204, A:M205, A:K220, A:G221, A:E222, A:T223, A:A224, A:D237, A:T238, A:S239, A:K240, A:V241, A:N242, A:P249, A:T250, A:F251, A:K252, A:G253, A:Q254, A:P255, A:Q356, A:T357, A:V358, A:D359, A:A360, A:A363, A:Q366, A:T367 | 89 | 0.627 |
| Protein Tax 1 | A:L2, A:F3, A:L6, A:S7, A:P8, A:L9, A:S10, A:S69, A:P70, A:S71, A:L72, A:P73, A:I74, A:T75, A:M76, A:F78, A:P79, A:A80, A:R81, A:R83, A:L85, A:P86, A:K88, A:A89, A:P90, A:S91, A:P93 | 27 | 0.663 |
|  | A:L12, A:L22, A:P23, A:P24, A:S25, A:D26, A:V27, A:S28, A:G29, A:L30, A:L31, A:L32, A:R33, A:P34, A:P35, A:P36, A:A37, A:P38, A:C39, A:L40, A:L41, A:L42, A:F43, A:L44, A:P45, A:F46, A:Q47, A:I48, A:S50, A:G51, A:L52, A:L55 | 32 | 0.56 |
