# Supplementary material for: Rational design of chimeric Multiepitope Based Vaccine (MEBV) against human T-cell lymphotropic virus type 1: An integrated vaccine informatics and molecular docking based approach
Source: PLoS One. 2021 Oct 27;16(10):e0258443. doi: 10.1371/journal.pone.0258443 (PMC8550388; doi:10.1371/journal.pone.0258443)
Supplement: S9 Table — (DOCX) [file pone.0258443.s013.docx]

**Table S9**: Disulphide bonds of MEBV construct predicted by Disulphide by design v2.2

| Res1 Amino acid number | Res1 Amino acid three letter code | Res2 Amino acid number | Res2 Amino acid  three letter code | Chi3 | Energy |
| --- | --- | --- | --- | --- | --- |
| 1 | GLY | 4 | ASN | -102.57 | 5.66 |
| 13 | VAL | 18 | CYS | 117.01 | 6.71 |
| 15 | GLY | 18 | CYS | 76.62 | 4.87 |
| 26 | LYS | 29 | GLN | -77.32 | 3.3 |
| 53 | PRO | 56 | LEU | 105.43 | 1.48 |
| 69 | THR | 355 | GLY | -109.28 | 3.79 |
| 71 | ARG | 357 | ASP | 120.88 | 3.14 |
| 75 | ARG | 360 | PHE | 116.36 | 2.66 |
| 79 | ALA | 373 | TRP | 100 | 4.65 |
| 90 | TRP | 382 | GLY | -71.13 | 1.42 |
| 113 | THR | 358 | LEU | -102.84 | 5.71 |
| 114 | LEU | 196 | LEU | -83.93 | 4.71 |
| 133 | GLY | 136 | LEU | -66.02 | 6.41 |
| 152 | LEU | 155 | TYR | -79.38 | 5.12 |
| 162 | THR | 182 | ASN | 82.44 | 4.54 |
| 164 | PRO | 180 | LEU | 122.27 | 7.64 |
| 168 | ALA | 176 | PRO | 77.01 | 5.56 |
| 178 | SER | 325 | CYS | -116 | 5.28 |
| 191 | ALA | 301 | CYS | -61.43 | 5.87 |
| 212 | CYS | 256 | ASP | 124.21 | 6.57 |
| 229 | ASN | 257 | ARG | -81.03 | 6.81 |
| 237 | ASP | 249 | TYR | 126.65 | 4.51 |
| 277 | PRO | 282 | LEU | 125.27 | 4.38 |
| 316 | LEU | 322 | ASN | 124.3 | 4.31 |
| 365 | GLY | 369 | HIS | 110.8 | 1.23 |
