# Supplementary material for: Rational design of chimeric Multiepitope Based Vaccine (MEBV) against human T-cell lymphotropic virus type 1: An integrated vaccine informatics and molecular docking based approach
Source: PLoS One. 2021 Oct 27;16(10):e0258443. doi: 10.1371/journal.pone.0258443 (PMC8550388; doi:10.1371/journal.pone.0258443)
Supplement: S10 Table — (DOCX) [file pone.0258443.s014.docx]

S10 Table: Linear B cell epitopes of MEBV predicted by ABCPRED server

| **Sequence** | **Position** | **Antigenicity** |
| --- | --- | --- |
| YTCIVCIDRASLSG | 230 | **0.7840** |
| LSPPITWPLLPAAY | 157 | **0.7215** |
| YTCIVCIDRASLST | 249 | **0.5929** |
| PFQIGPGPGPPAPC | 199 | **0.8883** |
| IGKCSTRGRKCCRR | 30 | **1.2783** |
| GGLCSKKPCLLLFL | 298 | **0.9185** |
| LPKEEQIGKCSTRG | 24 | **0.7537** |
| PITMRFPARWRAAY | 67 | **0.6089** |
| PGPGTNYTCIVCID | 224 | **0.7902** |
| CVQGDWCPISGGLG | 270 | **1.5881** |
| LGPGPGCVQGDWCP | 282 | **1.1846** |
| WRAAYFPARWRFLP | 76 | **1.4393** |
| LSGKKTNYTCIVCI | 316 | **1.9555** |
| GLDLLAAYLPSRVR | 133 | **0.6085** |
| TRGRKCCRRKKEAA | 35 | **1.0892** |
| RFLPWKAAAYPYWK | 86 | **0.8692** |
| TALLLFAAYLPITM | 57 | **0.5616** |
| YSLAAYQLSPPITW | 150 | **0.8471** |
| AKLSPLALTALLLF | 49 | **1.2360** |
| YAAQNRRGLDLLAA | 126 | **0.9610** |
| AYLPSRVRYPHYSL | 139 | **0.9232** |
| TWPLLPAAYEYTNI | 162 | **1.1029** |
| LPFQILSGKKTNYT | 331 | **1.4555** |
| TLPFNWTHCFAAYY | 113 | **0.7741** |
| HDVNFTAAYHLTLP | 102 | **1.0189** |
| AAYPYWKFQHDVNF | 93 | **0.8914** |
| VRYPHYSLAAYQLS | 145 | **0.9184** |
| NYTCIVCIDRASLK | 322 | **1.1782** |
| CFAAYYAAQNRRGL | 121 | **0.7025** |
| RKKEAAAKLSPLAL | 43 | **1.2657** |
| CLLLFLPFQILSGK | 306 | **0.7415** |
| AAYHLTLPFNWTHC | 108 | **1.0417** |
