# Supplementary material for: Rational design of chimeric Multiepitope Based Vaccine (MEBV) against human T-cell lymphotropic virus type 1: An integrated vaccine informatics and molecular docking based approach
Source: PLoS One. 2021 Oct 27;16(10):e0258443. doi: 10.1371/journal.pone.0258443 (PMC8550388; doi:10.1371/journal.pone.0258443)
Supplement: S11 Table — (DOCX) [file pone.0258443.s015.docx]

**S11 Table:** List of Conformational Epitopes of the final MEBV Construct

| **Sr No** | **Residues** | **No. of Residues** | **Score** |
| --- | --- | --- | --- |
| **1** | _:C11, _:R12, _:V13, _:R14, _:G15, _:G16, _:R17, _:C18, _:A19, _:V20, _:L21, _:S22, _:C23, _:L24, _:P25, _:K26, _:E27, _:E28, _:Q29, _:I30, _:G31, _:K32, _:C33, _:S34, _:T35, _:R36, _:G37, _:R38, _:K39, _:C40, _:C41, _:R42, _:R43, _:K44, _:K45, _:E46, _:A47, _:A48, _:A49, _:L51, _:S52 | **41** | 0.798 |
| **2** | :A83, _:R84, _:R86, _:F87, _:L88, _:P89, _:W90, _:A92, _:A93, _:A94, _:Y95, _:P96, _:Y97, _:K99, _:Y172, _:I376, _:D377, _:G378, _:R379, _:V380, _:I381, _:G382 | **22** | 0.777 |
| **3** | _:G1, _:I2, _:I3, _:N4, _:T5, _:L6, _:Q7, _:K8, _:Y9, _:Y10 | **10** | 0.762 |
| **4** | _:A78, _:Y147, _:P148, _:H149, _:Y150, _:S151, _:L152, _:A153, _:A154, _:Y155, _:Q156, _:L157, _:S158, _:P159, _:P160, _:I161, _:A332, _:S333, _:L334, _:K335, _:L337, _:T338, _:L339, _:P340, _:F341, _:N342, _:E362, _:Q363, _:G364, _:G365, _:L366, _:K367, _:K368, _:H369, _:Q370, _:I371 | **36** | 0.718 |
| **5** | _:I236, _:D237, _:R238, _:A239, _:S240, _:L241, _:S242, _:G243, _:P244, _:G245, _:P246, _:G247, _:N248, _:W275, _:C276, _:P277, _:I278, _:S279, _:G280, _:G281, _:L282, _:G283, _:P284, _:G285, _:P286, _:Q290 | **26** | 0.711 |
| **6** | _:T228, _:N229, _:T231, _:R257, _:A258, _:S259, _:L260, _:S261, _:T262, _:G263, _:P264, _:G265, _:P266, _:G267, _:G268, _:D269, _:C270, _:V271, _:Q272, _:G273, _:D274 | **21** | 0.681 |
| **7** | _:N182, _:G183, _:P184, _:G185, _:P186, _:G187, _:P188, _:P189, _:P190, _:A191, _:G299, _:L300, _:C301, _:S302, _:K303, _:K304, _:P305, _:C306, _:R331 | **19** | 0.632 |
| **8** | _:R131, _:G133, _:D135, _:L136, _:A138, _:A139, _:Y140, _:L141, _:P142, _:S143, _:R144 | **11** | 0.575 |
| **9** | _:Q129, _:N130, _:R132 | **3** | 0.551 |
| **10** | _:P67, _:I68, _:F347, _:P349, _:Q350, _:K352, _:R353 | **7** | 0.532 |
